# A scoping review on the psychosocial interventions used in day care service for people living with dementia

**DOI:** 10.1371/journal.pone.0295507

**Published:** 2023-12-11

**Authors:** Dympna Tuohy, Liz Kingston, Eileen Carey, Margaret Graham, Liz Dore, Owen Doody

**Affiliations:** 1 Department of Nursing and Midwifery, Health Research Institute, University of Limerick, Limerick, Ireland; 2 Department of Nursing and Midwifery, Health Research Institute, Health Implementation Science and Technology Cluster, University of Limerick, Limerick, Ireland; 3 Department of Nursing and Midwifery, University of Limerick, Limerick, Ireland; 4 Research Services Department, Glucksman Library, University of Limerick, Limerick, Ireland; Flinders University, AUSTRALIA

## Abstract

**Background:**

Adult Day care centres provide an important aspect of care provision through all phases of the dementia illness from diagnosis to the end of life (Dabelko HI 2008) supporting the well-being of both older people living with dementia and their care partners. Services within adult day care settings are designed to provide biopsychosocial health benefits to participants as well as care partner respite.

**Objective:**

To examine research studies, literature reviews and grey literature and identify and map the literature on psychosocial interventions used in day care services for older people living with dementia and chart their use, evaluation and outcomes. The research review question is “what are the psychosocial interventions used in day care service for older people living with dementia?” Psychosocial interventions are important non-pharmacological interventions which support people’s wellbeing.

**Methods:**

Inclusion/Exclusion criteria were identified and guided the search strategy. Participants were people aged 60 years and over living with dementia attending day care services. The use of psychosocial interventions for this cohort was the focus of the review. Databases were searched (Cochrane Reviews, CINAHL, Embase, Medline EBSCO, Medline Ovid, Medline PubMed, PsycINFO, Scopus, Open Grey, Lenus and WHO Global Index Medicus databases) using keywords/terms with Boolean operators from 2011 to 2023. Rayyan was used to extract and manage the data.

**Results:**

The findings present a narrative and charting of the data from the 45 papers that met the review criteria, and this data is mapped onto the five objectives. Within this review, interventions were grouped into five broad types: nature (n = 6 papers), memory/cognitive (n = 11 papers), social (n = 17 papers), animal (n = 4 papers), or physical/sensory (n = 7 papers) based interventions.

**Conclusions:**

This review has illustrated the wide variety in the types, range and facilitation of psychosocial interventions within adult day care services. This review highlights the potential benefits of these interventions. However, findings must be considered in the context that many were provided as brief intervention studies with little evidence of continuation after the study and further research is required given the complex and diverse range of interventions. Results will be of interest to practitioners planning to implement or evaluate psychosocial interventions used in day care services for older people living with dementia.

## Introduction

Globally there are approximately 50 million people (majority of whom are older people) currently living with dementia, and this figure is set to double by 2030 and increase to 152 million by 2050 [1–3]. While dementia is not an normal part of ageing, it is recognised as affecting many older people. Dementia is an umbrella term used to describe a group of complex disorders characterized by progressive decline in cognitive functions [4].^.^Dementia, therefore is a global public health concern with the majority of diagnosis in people over 60 years. The majority of this population live in their own homes. The dementia illness trajectory can extend over several years. Traditionally, older people (people aged 60years and older) living with dementia were regarded as patients who were passive receivers of care. Care provision for people living with dementia has shifted from a paternalistic perspective, to a view where older people living with dementia are now regarded as being active users of healthcare services with diverse health care needs [5].^.^ Consequently, a diverse range of interventions have been designed and implemented aiming to support the wellbeing of people with dementia and their care partners. Specifically, community based adult day care services provide an important aspect of care provision along the care continuum, supporting the well-being of both older people living with dementia and their caregivers [6].

In general, day centres or day care centres are known internationally to provide a range of educational, health, social and rehabilitative services for people with disabilities, across the lifespan. Such services can be governmental, voluntary or privately run organisations, with access though either personal choice of the individual or by referral. Eligibility conditions vary. More specifically, day care services with a diverse range of interventions have been designed and implemented to support the wellbeing of people living with dementia and their care partners. These adult day care services involve an integrated system delivered over a period of time to older persons living with dementia, supporting physical, emotional, mental, social, environmental and spiritual health and wellbeing of both the older person living with dementia and their care partner(s) across all phases of the dementia illness from diagnosis to the end of life [6].

Day care services are designed to provide biopsychosocial health benefits to participants as well as care partner respite. While there are various definitions of day care, all share common goals [7–9]. Essentially ‘day care center’ is a generic term describing community (building-based and green farms) services that offer a wide variety of programmes and services. Within this review, day care centers are defined as community based services that provide care and/or health-related services and/or activities specifically for older people irrelevant of whether these individuals reside in affiliated residential centres or at home and in which people can attend for a whole day or part of a day [7–9] The objectives of day care are to: improve quality of life through creating a meaningful day within a safe environment; focus on social interaction through a variety of activities organized individually or in groups; provide people with the support to continue living at home; provide respite for carers and for some older people, postpone the need for nursing home placement [8, 10, 11]. Research has shown the benefits of day care for older people living with dementia [12, 13]. and their families [14–17]. Currently evidence based interventions for dementia care comprise both pharmacological and non-pharmacological strategies and methods.

Increasingly there has been an emphasis on non-pharmacological interventions to support people living with dementia specifically psychosocial interventions. Olazarán et al. [18] describe a wide range of non-pharmacological therapies in Alzheimer’s disease including, cognitive therapies, reminiscence, music, therapeutic touch, light, physical exercise and development coping skills. McDermott et al. [19] outline psychosocial interventions as cognitive and physical as well as interventions which include social and psychological components. Social engagement and the social activities that are provided include psychosocial interventions. Psychosocial interventions can be defined as “interpersonal or informational activities, strategies or techniques that can target biological, behavioural, cognitive, emotional, interpersonal, social or environmental factors with the aim of improving an individual’s health functioning and mental well-being” [20] These are provided in a variety of health care centres including adult day care centres. Within the context of the diversity of psychosocial interventions being used in day care provision, it is important to identify the type, range and use of psychosocial interventions currently being used. This scoping review aims to do this. Specifically, the research review question is “what are the psychosocial interventions used in day care service for older people living with dementia?”

## Methods

The six-stage framework by Arksey and O’Malley [21] and further developments by several authors [22–25] framed this review. Members of the research team represent expertise in nursing, older person, methodological and library/information. The review is reported in line with the Preferred Reporting Items for Systematic Reviews and Meta-Analyses Extension for Scoping Reviews [26]. **In stage 1**: we identified the research question, “what are the psychosocial interventions used in day care service for older people living with dementia?”. Discussions within the research team focused on the need to scope and review the existing literature with the aim of identifying the type, range and use of psychosocial interventions in the delivery of evidence based care in day care service for older people living with dementia. There are five objectives:

1. What are the types and range of psychosocial interventions used in day care services?
2. What is the reported use and facilitation of psychosocial interventions?
3. What are the evaluation methods used for psychosocial interventions?
4. What are the reported psychosocial interventions outcomes?
5. What are the reported adaptations to psychosocial interventions?

**Fig 1:**
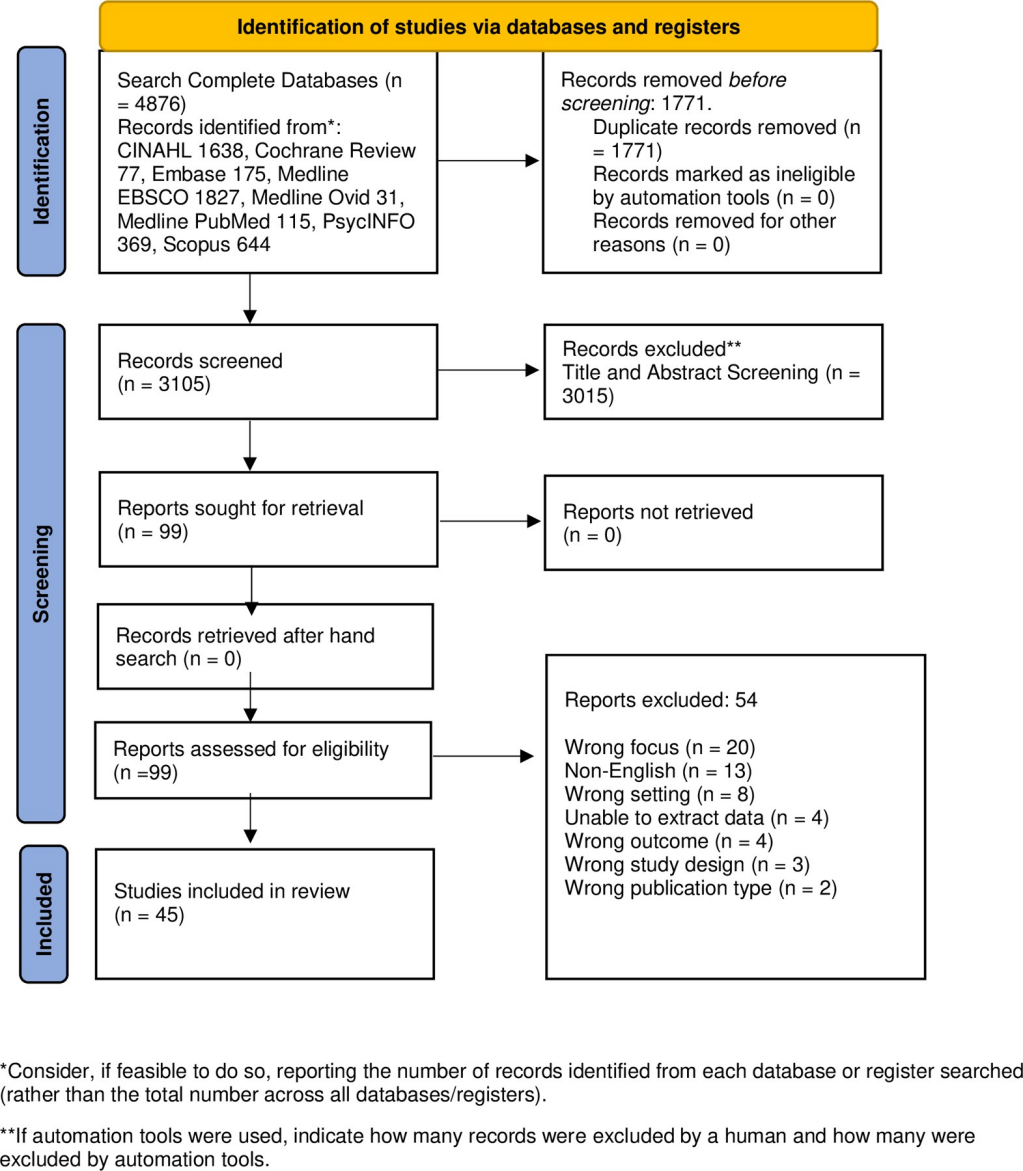
Prisma flow chart.

**Stage 2**: searching for relevant studies was guided by the PCC format process where participants were people aged 60 years and over living with dementia (Participant), attending a day care centre (Concept) and receiving a psychosocial intervention (Context). Both databases and grey literature searches were performed in December 2021 and updated in February 2023 of the following: Cochrane Reviews, CINAHL, Embase, Medline EBSCO, Medline Ovid, Medline PubMed, PsycINFO, Scopus, using keywords/terms with Boolean operators (S1 File: search strategy). A grey literature search was undertaken, using the same search terms, inclusion and exclusion criteria in Open Grey, Lenus and WHO Global Index Medicus databases. All results were exported to endnote and duplicates removed and for screening and voting purposes exported to Rayyan. The search and screening process are reported using the PRISMA flow chart [27] (Fig 1: Prisma flow chart). **Stage 3**: selecting studies, after duplicates were removed the remaining independent screening was undertaken by two reviewers against the inclusion criteria presented in Table 1. Initial screening was by title and abstract and then at full text. Conflicts were resolved by involving a third reviewer with consensus reached. **Stage 4**: charting the data, data were extracted from all studies meeting the inclusion criteria into a predetermined data extraction table (S2 File: data extraction table (DET)), addressing details pertaining to authors, year and country, aim, design, type of intervention, use and facilitation of the intervention, outcomes and reported adaptations. **Stage 5**: collating, summarizing, and reporting the results. The extracted data were charted and reported descriptively with results mapped and presented in relation to the review questions. The results were presented in descriptive form using the sub-questions as headings and as appropriate tables and diagrams were used to illustrate the findings, these were enhanced by narrative text. In line with the objective of scoping reviews the results assisted in making recommendations. **Stage 6:** consultation, this is an optional step and not utilized in this review given the existing experience and expertise of the author group and difficulty in recruiting experts by experience when the project initially commenced during COVID-19.

**Table 1:** Inclusion/exclusion criteria.

Criteria	Inclusion	Exclusion
Time frame	2011–2023	Before 2011
Language	English	Not English
Types of Literature Sources	• Research (qualitative, quantitative and mixed design studies) • Reviews (systematic, integrative and narrative) • Grey Literature (national and international policies, strategies, guidelines and standards)	Grey literature other than national and international policies, strategies, guidelines and standards.
Participants	People aged 60 years and over, living with dementia attending day care.	Non day care centres e.g. residential care Participants younger than 60years
Concept/interventions	Psychosocial interventions used in day care	Not psychosocial interventions
Context	Use of psychosocial interventions used within a day care setting for older people (living with dementia) in primary care within an international context.	Non-use of psychosocial interventions in day care setting within an international context.

## Findings

The findings present a narrative and charting of the data from the 45 papers that met the review criteria and this data is mapped onto the five objectives outlined in step one of the framework [21] Within this review, interventions were grouped into five broad types: nature (n = 6 papers), memory/cognitive (n = 11 papers), social (n = 17 papers), animal (n = 4 papers), or physical/sensory (n = 7 papers) based interventions.

### Types and range of psychosocial interventions used in day care service

Several different types of psychosocial interventions were identified as a single or combined interventions (Table 2). Nature based interventions representing the person’s experience or understanding of their own natural world included: green care farm [28, 29], farm based [30, 31] garden based [32] meaning in life in general [33].Memory/cognitive-based interventions related to reminiscence structured sessions [34, 35], life story interviews [36], life review programme[37], life story work [38], emotional therapy [39] and cognitive behavioural therapy [40, 41] Combined approaches included combined cognitive training, music and art therapy [42] combined environmental barriers and cognitive behavioural therapy [43] and combined reality orientation and cognitive behavioural therapies [44].

**Table 2:** Types and source of interventions.

Psychosocial intervention:	Paper
**Nature based interventions**	
Life story/review/reminiscence	Chang *et al*., 2018; Gregory, 2011; Van Bogaert, 2013; Lin *et al*., 2011; Novy, 2018
Nature/garden/farm based	De Bruin *et al*. 2012; De Bruin *et a*.*l*, 2015; De Bruin *et al*. 2021 ; Finnanger *et al*. 2020; Ibsen & Eriksen 2021; Noone & Jenkin 2018
**Memory/Cognitive based interventions**	
Emotional/cognitive based	Maeda *et al*. 2016; Kwon et al. 2020; Kallio et al. 2021 ; Jung *et al*., 2020; Padilla *et al*., 2013; Salotti *et al*. 2013
**Social based interventions**	
Music based	Baker & Stretton-Smith, 2017; Osman et al., 2016; Ihara et al., 2018 ; Peeters et al. 2016
Activity based	Gjernes, 2017; Westcott, 2011; Thompson and Fletcher, 2019; Lancioni et al., 2015; Cheung et al. 2019; Jaana, 2020; Rokstad 2019; van Haeften-van Dijk et al. 2017; Ching-Teng, 2019; Hattford-Letchfield, 2012; Shoesmith et al., 2020
Technological activity	Soler et al., 2015; Smith et al., 2020
**Animal based interventions**	
Animal assisted	Olsen et al., 2016; Olsen et al., 2019; Sánchez-Valdéon et al. 2019; Dabelko-Schoeny et al. 2014
**Physical/Sensory based interventions**	
Multisensory	Riley-Doucet and Dunn, 2013; Lorusso and Bosch 2018; Moorman et al. 2017
Movement based	Rylatt, 2012; Aguinaga & Marquez, 2019; Chang et al. 2011; Karania, 2017

The social based interventions related to a single or range of activity combinations. Single social activities related to board games [45] comedy workshops [46] humanoid robot [47], adult care programme [14, 48, 49], technological group activity [50] and art activities [51] Combined social interventions were utilised in five studies [52–56] and within two of these studies occupational activities were incorporated [52, 55] In addition, the broad activity of music was identified and included song writing [57] listening to music to stimulate memory [58] ‘singing for the brain’ [59] and the development of a music application prototype to assist in human-computer interaction [60] Animal-based interventions related to the use of animal assist (canine) therapy [61–63] and an equine-assisted therapy intervention [64]. Physical/sensory-based interventions evident within this review included multisensory environments [65, 66], aromatherapy [67] dance activity [68], exercise programmes [69, 70]., while Rylatt [71] utilised a creative therapy exercise programme incorporating a combination of interventions (dance, drama, music, movement).

### Use and facilitation of psychosocial interventions

Within the facilitation of psychosocial interventions several were ongoing while others are facilitated within a specific period/time limited specific programmes. Each study within the five broad types: nature, memory/cognitive, social, animal, or physical/sensory based interventions are presented in Table 3.

**Table 3:** Use and facilitation of psychosocial interventions.

Author	Activity facilitated
**Nature based interventions**	
De Bruin et al 2012	Attendance at green care farms versus attending regular day care farms.
De Bruin et al. 2015	Standard participation green care farms.
De Bruin et al. 2021	Broad description of nature-based adult day services, non-specific.
Finnager et al. 2020	The use of farm-based day care to promote physical functioning, functional mobility and physical activity.
Ibsen & Eriksen, 2021	Farm-based day care service from May and November, structure of the day included breakfast, work session, coffee break, walk or other activities and then dinner before leaving.
Noone & Jenkin, 2018	Gardening session, weekly session over six weeks.
Novy, C. 2018	Life story project was a complex intervention that included a phase of individual work for the purpose of recording a life story, followed by an interactive performance of this story in a group setting. Facilitated by 12 storytellers over two years. Life story recorded and performed by utilizing a narrative, centering the person, picturing the story, recruiting an audience, dramatizing the story, and performing the life story.
Chang et al. 2018	Six group sessions of reminiscence therapy, one hour each, over a six-week period. The topics for the sessions were generated from Life Story interviews of 1–2 hours held once a week for a month prior to the commencement of the study. Individual sessions using general reminiscence approach identical for individual and group sessions. Primary researcher, specialized in dementia care and reminiscence therapy, conducted the intervention and led group activities. Two research assistants served as group co-leaders who observed interactions among group members, encourage group discussion, and support participants’ emotions during activities and record participants’ emotional and behavioural changes. In addition to the research assistants, two staff familiar to the participants from the day care center assisted with each section to facilitate discussion and help participants when needed.
Gregory, 2011	Try to Remember Intervention consisted of three core components: reminiscence sessions with service users; readback sessions with service users, family members, care staff and other health care professionals, such as local GPs; and skills training workshops with care staff. Delivered over four weeks and sessions averaged 40 minutes with clients typically seen 2/3 times. Readback sessions lasted around 50 min and two/three-hour workshops were held: listening skills; non-verbal communication; writing skills; and boredom, anxiety and isolation amongst clients.
Van Bogaert et al. 2013	Structured reminiscence sessions based on SolCos reminiscence model over 4 weeks, 1 facilitator.
Lin et al. 2011	Revised Life Review Programme LRP-TW Erikson’s life stages each session had a specific life theme in sequence, activities included singing Taiwanese lullaby songs, drawing lots, playing with traditional toys (e.g., bamboo dragonfly, catapult, sandbag, etc.), enjoying a traditional puppet show and Taiwanese opera, listening to or watching old Taiwanese records and movies, doing garden activities, and enjoying the traditional tea ritual. The primary researcher, conducted the intervention, delivered over 10 successive sessions over 2 weeks. Two to three staff from the center assisted as facilitators. One session was administered for 60 minutes each weekday during the intervention period. Each session had a specific life theme in sequence, including trust-mistrust in infancy, autonomy-shame in toddlerhood age, initiative-guilt in play age, Industry-inferiority in school age, identity-confusion in adolescence age, intimacy-isolation in young adulthood, generativity-self-absorption in middle adulthood, and integrity-despair in old age, plus the first session as an introduction and the last session as a celebration. This framework guided the selection of relevant activities based on how well each activity evoked memories related to target themes; how well each activity matched the life stage targeted.
**Memory/Cognitive based interventions**	
Kwon, et al., 2020	Cognitive training programme, two groups (cognitive training and standard clinical care, standard clinical care). 3 hours a day, 5 days a week for 12 months. Instructor an experienced clinical neuropsychologist delivering attention training (paper or computer assisted attention training), memory training (training in the recall of a list and remembering the location of objects in the room), visuoconstruction training (drawing various things and changing blocks), physical training (massed calisthenics), occupational training (creative activity such as drawing or knitting), and speech training.
Kallio, et al. 2021	Cognitive training programme based on paper-and-pencil tasks with cognition as a primary target. Two groups (systematic CT, attended day care as usual) 45 minutes twice a week over 12 weeks. Trained psychology students administered CT under the guidance and supervision of an experienced neuropsychologist. Each session included cognitive exercises from four task categories: visuomotor, perceptual, conceptual, and interactive tasks (simple word or card games). Interactive tasks were included at the end of each session for up to 10 min to keep up the participants’ training motivation.
Jung et al. 2020	Integrated cognitive integrated therapy programme comprising cognitive training, music therapy, art therapy. 16 sessions of 60 minutes each conducted over 8 weeks (twice weekly). Cognitive training comprised of 5 stages: introductory activity, brain health lifestyle education, activity briefing, main activity, and finishing activity. The music therapy program was comprised one 60-minute session per week that was conducted for a total of 8 weeks. This program involved active music activities split into three phases: introductory activity, main activity, and finishing activity. The art therapy program comprised one 60-minute session per week that was conducted for the duration of 8 weeks.
Padilla et al. 2013	Environmental (subjective barriers), cognitive/behavioural (cognitive training with differential reinforcement) and combined (subjective barriers + cognitive/behavioural). 33 sessions of four hours. Environmental intervention the exit door was altered (eight strips of black tape were placed 25cm from the exit door with 4 cm distance between and four other strips placed on the glass door 25 cm from the floor. Cognitive/behavioral intervention consisted in differential reinforcement of behaviors while working with the patient on different types of language, memory, and attention tasks which were used to distract the person’s attention from the door.
Salotti et al. 2013	Reality orientation therapy and cognitive training constituted cognitive rehabilitation for the experimental group and the control group has a specific stimulation. All participants underwent a series of specific group stimulations 3 days a week. The experimental group also underwent cognitive stimulation based on a combination of two therapies ROT and CT three times a week.
Maeda, et al., 2016	Dramatic emotional therapy programs carried out for approximately one hour. Activities chorus with guitar, volleyball using balloon, line drawing for coloring, karaoke, bowling game watching TV program of popular song.
**Social based interventions**	
Gjernes, 2017	Social activity, knitting. Researcher participated in organized activities some participants attended 5 days a week, others came 2/3 days a week. Knitting activity organized by staff members to help them create a social group. The knitted elements formed a complete knitted product and then sold.
Westcott 2011	Recreational groupwork included a variety of activities such as bingo, scrabble, word search and poems, initially aimed at one specific individual. Group not static but generally 2–6 members with 1 to 3 staff.
Thompson & Fletcher 2019	General and included physical care provision, the use of social interactions and the development of social relationships. Not specifically outlined all within the whole programme, so not explicate as to when/how specific psycho-social interventions were focused upon.
Lancioni et al., 2015	Self-care, grooming, leisure, music, occupational activities. Computer-aided sessions as well as baseline and control sessions, lasted 5 min. Computer presenting photos and videos and providing encouragements to talk as well as attention and guidance. Sessions occurred per day (on an individual basis). Sessions were video-recorded and then scored by a research assistant.
Baker & Stretton-Smith, 2017	Ten one-hour songwriting sessions were facilitated once a week by two music therapists, one author and another unaffiliated registered music therapist. Leadership changed fluidly as needed throughout each session, with one music therapist leading the songwriting experience while the second music therapist supported the leader.
Osman et al. 2016	Singing for the brain group activity combines reminiscence therapy and music, involving a musician. Gather in a large circle and follow instruction from the musician. The session starts with warm-up exercises for voice and body before moving on to singing familiar songs that follow a different theme each week. Attendees are provided with song sheets including the lyrics for each song. Songs are sung in unison accompanied by the musician or in rounds with harmonies. Depending on access to equipment, attendees can use percussive instruments during the session. At the beginning and end of each session, there is an opportunity for refreshments and time to socialise.
Ihara et al. 2018	An individualized music listening system/program. Individuals were given an iPod programmed with their personalized playlist. Assess changes in mood and agitation.
Peeters et al. 2016	Music application prototype where the person with dementia and relative were paired to use the prototype during an evaluation day.
Ching-Teng 2019	The board game sessions were conducted for 90 min each for a total of 12 weeks, led by a recreational therapist. Before the activity, the physical and mental states of participants were examined, and the social worker ensured the safety of the participants during the activities. Various board games were selected for the sessions.
Hattford-Letchfield, T. 2012	Comedy activities workshops using improvisatory activities and comedy, four workshops lasting 2/3 hours each ran weekly for 4 weeks. Workshop 1: Grangers and staff receive the news that the Queen is planning to visit their day centre and much excitement and planning begins. Workshop 2: the day centre makes practical preparation for the Queen’s visit. Workshop 3: Grangers prepare their entertainment for the Queen’s visit. Workshop 4: Her Majesty the Queen visits the Grange.
Soler et al. 2015	A humanoid robot, who is able to use oral language and move like a human; an animal-shaped robot, who does not use oral language but sounds and moves like an animal; and conventional therapy. Phase 2 a trained dog; an animal-shaped robot and with conventional therapy. Therapy sessions were held 2 days per week over 3 months. All therapeutic sessions were conducted by the same therapist, with the same structure as the other therapeutic programs, at the same time of day and for the same duration of time (30–40 min). The therapists were certified occupational and physical therapists, and neuropsychologists, they received instructions on the implementation and possible uses of robots and animals as they had no previous expertise in this area. The animal therapists and robot engineers did not participate in the therapy; they monitored session from one side of the room, out of the patients’ view. Session guides were written and followed in every session.
Rokstad 2019	Not described
Jaana, M. 2020	Adult day program enabling structured and proactive communication with caregivers and members of the care team in the community. A coordinator assigned to each senior client/caregiver. The coordinator conducts weekly follow-ups with the caregiver and provides support and referrals on a need basis when issues arise with the senior client with dementia. 50 weeks per year, 3 days a week. Sessions, involving fitness fun and cognitive activity, until noon when they have lunch followed by a physical activity session is organized followed by a theme presentation.
van Haeften-van Dijk et al. 2017	Not described
Smith et al. 2020	Technology facilitated group activity sessions, twice a week for a period of four weeks and all were video recorded. The researcher uploaded the tablet computers with familiar activities already enjoyed by members that appeared to effectively transfer from their traditional form to technology formats, including dominos, solitaire and jigsaws. New interactive applications were also uploaded including the keyboard, guitar and drum kit.
Shoesmith et al. 2020	Structured, person-centred session each week. Different activity inspired by a different theme, involves a variety of materials (paints, ink, pencils, fabric, 3D materials), techniques (brush, paint roller, pipettes) and final debriefing before finishing group aimed towards stimulating participant choice and independence. The intervention delivered over six weeks, one- hour sessions, co-facilitated by two trained facilitators.
Cheung et al. 2019	Eight weekly group-based sessions of the CoS-Play, each 45 to 60 minutes in length. Content of each session activities comprising 6 elements: visuospatial and fine motor activities (making handicrafts), kinesthetic and gross motor activities (batting a balloon), language and verbal activities (telling stories), executive function activities (card games), auditory music and rhythmic activities (playing percussive musical instruments), and social interaction. Control and intervention group. The control group took part in social activities (reading newspapers, watching television) in a similar pattern as with the intervention group.
**Animal based interventions**	
Olsen et al. 2019	Animal assisted activity intervention consisted of 30-min sessions twice per week for 12 weeks. Sessions led by a qualified dog handler. Every session started with a greeting round, where each participant got to pet the dog and give it treats. The handler then started the different activities, which could be petting the dog, giving the dog a treat or throwing a toy for the dog to fetch. A health care worker was present during all sessions. The dogs were kept on/off lead, depending on interaction. No dogs were forced to do anything they were not comfortable doing. No activities were mandatory for the participants.
Olsen et al. 2016	Animal assisted activity led by qualified dog handler, 30-minute sessions twice a week for 12 weeks.
Sánchez-Valdéon et al. 2019	Canine therapy, dog specifically trained by a canine specialist and intervention carried out by a professional trained in canine-assisted therapy. Session 30 minutes per week for 12 months. Programme included a mix of guided motion exercises, activities and animal to human interaction for improving affective state.
Dabelko-Schoeny et al. 2014	Equine-assisted therapy intervention program occurred once a week for four weeks and consisted of three specific intervention activities. Each activity was a 15-minute hands-on experience with the horse followed by 5 minutes of rotation to the next activity. Activities included, grooming, observation of horse-to-horse interactions and interaction with the horse, leading the horse around the arena and placing and removing a halter, painting with symbols, and washing and reward feeding horses.
**Physical/Sensory based interventions**	
Riley-Doucet and Dunn 2013	Multisensory environment 4-week period (average 5 times a week and average 25 minutes per session) utilizing the following sensory equipment, aroma diffuse, chase light string, evening breeze, fibreoptic string light, plasma ball, solar effects projector, stereo and speakers (music) and vibrating tube.
Lorusso & Bosch, 2018	Systematic literature review of multisensory environments which sought articles for review that incorporated at least three sensory-based items including ergonomic virbroacoustic furniture, bubble tubes, color-changing lights, music, and fiber optics. Some studies compared effects of sessions, some studies offered sessions at regular intervals during the week, same time daily and same duration each week, others administered therapy intermittently, some studies administered specifically when BPSD occurred. Overall, the approach to data collection varied widely between 2 weeks to 1 year, and intervention sessions lasted between 8 and 40 minutes.
Moorman et al. 2017	Aromatherapy over a four-month with 20 min twice a day, once in the morning and once in the mid afternoon during active clinic days.
Rylatt 2012	Creative therapy activities including dance, drama, music and movement. Implemented over an eight-week period, three times a week, with each session lasting approximately 30 minutes.
Aguinaga & Marquez 2019	Modified dance programme called BAILAMOS, a 4-month, twice-weekly program. Dance session 1 hour includes four dance styles: Merengue, Cha Cha Cha, Bachata, and Salsa. Monthly discussion sessions are also held and research staff member was present at all dance sessions to set up the room and observe the class.
Chang et al. 2011	Exercise programme consisting of stretching and walking exercises five times per week, and leg-weight bearing three times per week for 20–30 min. In the morning, subjects were encouraged to walk 20–30 min while listening to familiar music. The walking exercise was preceded and followed by 5 min of stretching exercise to warm up and cool down. Those who regularly performed exercise were given positive feedback and encouragement.
Karania 2017	Gymnastics-based exercise programme, each session lasted 1 hour and delivered by the same person. One or two residents had a family member attend with them, and care home activity attended and occasionally other staff members. The delivery of each session was structured around eight core activity themes: meeting and greeting; gentle warm-up exercises; facial expressions and arms crossing the mid-body line; bilaterally asymmetrical movement patterns; paper, scissors, stone game; partner working; cognitive stimulation activity and relax and wind down exercise.

Participants attended a green care farm [28, 29, 33] and also a non-time limited programme with a similar structure for each day across all sites [31]. Whereas programmes focused on the use of GCFs to promote physical activities [30] and the use of a six-week programme of weekly gardening session [32]. Memory/cognitive-based interventions that were time limited ranged from five days [40], two weeks [37], four weeks [33, 34], six weeks [36], eight weeks [42] and 12 weeks [41]. Other programmes ran for over 12months [40, 44], over two years [38] or were open-ended [39]. essions ranged from one hour/day [39], three hours a day [40], one session per week [36], two/three sessions per week [34, 41, 43], four sessions per week [42], six/eight sessions per week [35], ten sessions per week [37] to thirty-three sessions per programme [43]. Facilitation was led by the primary researcher [36, 37] or a facilitator [34, 35, 38] but involved the inclusion of research assistants [36], staff [34, 37, 38] or another facilitator [35].

Social based interventions were individual sessions [55, 58, 60] or group sessions [52–54, 57, 59] The group programmes were open to whomever attended the centre on the day however, in one study six people participated in the original programme and ten in the modified programme [55]. Programmes ran for four weeks [46, 50], six weeks [51] eight weeks [56], three months [47] or appeared to be open-ended [45, 48]. Sessions ran for 45-60minutes [47, 56], one hour/day [51], two and half hours/day [48], two/three hours per week [46] or twice/week [50].

Two papers [14, 49] do not describe their use or facilitation while in other papers facilitation was supported by staff and therapists [57, 59]. Animal-based interventions were time limited ranging from one to two session per week over a period of four weeks [64], 12 weeks [61, 62] or 12 months [63]. Professional animal handlers were involved in all four programmes in conjunction with other facilitators [61–64]. Physical/sensory-based interventions were time limited ranging from four weeks [65] two months [67, 71], four months [68]. In contrast another review [66] reported time limits from 2 weeks to 12months while another two exercise programmes were not time limited [69, 70]. Sessions lasted up to 20 minutes [67], 30 minutes [65, 69–71] 40 minutes [66] to one hour/day [68].

### Reported evaluation methods

A range of evaluations methods were used for the psychosocial interventions and can be grouped under into four categories 1) instrument/measurement measures (a broad spectrum of bio-psycho-social intervention measures) 2) exercise/functional measures, 3) qualitative measures and 4) activity measures and mapped under the five broad types: nature-based memory/cognitive, social, animal, or physical/sensory based interventions identified in this review (Table 4). Five papers did not report outcome measures [14, 34, 49, 57, 70]. Within the first category (instrument/measurement) fifty outcome measures were identified across 26 papers. The mini-mental state examination was reported in eight papers [28, 35, 36, 40, 41, 43, 47, 61]. the Clinical Dementia Rating Scale in five papers. [30, 40, 41, 61, 63] The Geriatric Depression Scale [35, 42, 44, 45] and the Cornell Scale for Depression in Dementia [35, 58, 66, 69] in four papers. The Cohen-Mansfield Agitation [58, 66], the Frontal Assessment Battery [35, 41] the Montreal Cognitive Assessment [45, 56], the Neuropsychiatric Inventory [35, 47] and the Quality of Life in Late-Stage Dementia Scale [47, 63]^,^ in two papers.

**Table 4:** Evaluation methods: Outcome measures.

Evaluation method	Paper	Combined evaluation approach
**Nature based interventions**		
Mini-mental state examination, Cornell Scale for Depression in Dementia, Activity observation form, Video recording.	Chang *et al*., 2018	
Mini State Examination, Barthel Index, Interview for Deterioration in Daily living in Dementia, Interviewes.	De Bruin *et al*. 2012	
Interviews.	De Bruin *et al*., 2015	
Interviews.	De Bruin *et al*. 2021	
Actigraphs, Clinical Dementia Rating Scale, Timed Up and Go-test.	Finnanger *et al*. 2020	
Interviews	Ibsen & Eriksen 2021	
Not reported.	Gregory, 2011	
Mini Mental State Examination, Frontal Assessment Battery, Neuropsychiatirc Inventory, Geriatric Depression Scale, Cornell Scale for Depression in Dementia.	Van Bogaert, 2013	
Short Form Health Survey.	Lin *et al*., 2011	
Interviews, Observations.	Noone & Jenkin 2018	
Video, Observations, Reflective Discussions.	Novy, 2018	
**Memory/Cognitive based interventions**		
Emotional Satisfaction Index.	Maeda *et al*. 2016	
Mini-Mental State Examination, Clinical Dementia Rating Scale, Consortium to Establish a Registry for Alzheimer’s Disease ‐ Neuropsychological Battery.	Kwon et al. 2020	
Clinical Dementia Rating Scale, Mini-Mental State Examination, Alzheimer’s Disease Assessment Scale-Cognitive subscale, Frontal Assessment Battery, The Clock Drawing test, Trail Making Test, Digit Span task ‐ Finnish Wechsler Memory Scale.	Kallio et al. 2021	
Korean version ‐ Mini-Mental Status Examination, Korean Dementia Screening Questionnaire-Cognition, Geriatric Depression Scale, Beck Anxiety Inventory, Seoul-Instrumental Activities of Daily Living, Seoul Neuropsychological Screening Battery.	Jung *et al*., 2020	
Algase Scale.	Padilla *et al*., 2013	√
Milan Overall Dementia Assessment, Mini Mental State Examination, Geriatric Depression Scale.	Salotti *et al*. 2013	√
**Social based interventions**		
Observations	Gjernes, 2017	
Observations	Westcott, 2011	√
Interviews.	Thompson & Fletcher, 2019	√
Observations.	Lancioni et al., 2015	√
Montreal Cognitive Assessment, Verbal fluency, Interviews.	Cheung *et al*. 2019	√
Semi-structured interviews, observations.	Baker & Stretton-Smith, 2017	
Not reported.	Osman et al., 2016	
Cornell Scale for Depression in Dementia, Cohen-Mansfield Agitation Index	Ihara et al., 2018	
Observations, Interviews.	Peeters et al. 2016	
Geriatric Depression Scale, Montreal Cognitive Assessment Scale, Interview.	Ching-Teng, 2019	
Video, Photograph, Reflective Discussions.	Hattford-Letchfield, 2012	
Global Deterioration Scale, Severe Mini Mental State Examination, Mini Mental State Examination, Neuropsychiatric Inventory, Apathy Scale for Institutionalized Patients with Dementia Nursing Home version, Apathy Inventory, Quality of Life in Late-Stage Dementia Scale.	Soler *et al*., 2015	
Not reported.	Rokstad 2019	
Not reported.	van Haeften-van Dijk et al. 2017	
Caregivers’ survey, Instrumental Activities of Daily Living.	Jaana, 2020	
Video recordings.	Smith *et al*., 2020	
Quality of Life in Alzheimer’s Disease, Social Functioning in Dementia Scale, Lawton Scale of Activities of Daily Living, Interviews.	Shoesmith *et al*., 2020	
**Animal based interventions**		
Berg Balance Score, Quality of Life in Dementia Scale, Clinical Dementia Rating scale.	Olsen et al., 2016	
Mini-Mental Status Examination, Clinical Dementia Rating Scale, Video-recordings, An ethogram catalogue.	Olsen et al., 2019	
Quality of Life in Late-Stage Dementia Scale.	Sánchez-Valdéon et al. 2019	
Behavioral Observation Recording, Modified Philadelphia Geriatric Center Affect Rating Scale, Salivary cortisol, Modified Nursing Home Behavior Problem Scale.	Dabelko-Schoeny et al. 2014	
**Physical/Sensory based interventions**		
Observations, Agitated Behavior Scale, Caregiver Exit Survey.	Riley-Doucet and Dunn, 2013	
Cohen-Mansfield Agitation Index, Neuropsychiatric Inventory Nursing Home, Cornell Scale for Depression in Dementia, Psychotic Behavior Assessment Record, Daily Observation Scale, Clinical Global Impression-Improvement.	Lorusso and Bosch 2018	
Behavior Intervention Monthly Flow Record.	Moorman *et al*. 2017	
Tool designed to evaluate attendance, and outcomes of the creative therapy sessions in relation to creative self-expression, communication, pleasure and enjoyment, and general engagement.	Rylatt, 2012	
Accelerometer and inclinometer, Sedentary behaviour questionnaire, Non-exercise equation to calculate cardio-respiratory fitness.	Aguinaga & Marquez, 2019	
Recreational activities of daily living scale, Functional ability tests.	Chang *et al*. 2011	
Not reported	Karania, 2017	

The remaining measure were reported in one paper each: Agitated Behavior Scale [65], Algase Scale [43], Alzheimer’s Disease Assessment Scale-Cognitive subscale [41], An ethogram catalogue [62], Apathy Inventory Scale [47], Apathy Scale for Institutionalized Patients with Dementia Nursing Home version [47], Barthel Index [28], Beck Anxiety Inventory [42], Behavior Intervention Monthly Flow Record [67], Behavioral Observation Recording [64], Berg Balance Score [61], Caregiver Exit Survey [65], Caregivers’ Survey [48], Clinical Global Impression-Improvement [66], Consortium to Establish a Registry for Alzheimer’s Disease ‐ Neuropsychological Battery [40], Daily Observation Scale [66], Digit Span task ‐ Finnish Wechsler Memory Scale [41], Emotional Satisfaction Index [39], Global Deterioration Scale [47], Instrumental Activities of Daily Living [48], Interview for Deterioration in Daily living in Dementia [28], Korean Dementia Screening Questionnaire-Cognition [42], Korean version ‐ Mini-Mental Status Examination [42], Lawton Scale of Activities of Daily Living, [51] Milan Overall Dementia Assessment, [44] Modified Nursing Home Behavior Problem Scale [64], Modified Philadelphia Geriatric Center Affect Rating Scale [64], Neuropsychiatric Inventory Nursing Home [66], Psychotic Behavior Assessment Record [66], Quality of Life in Alzheimer’s Disease [51], Quality of Life in Dementia Scale [61], Recreational Activities of Daily Living Scale [69], Salivary cortisol [64], Sedentary behaviour questionnaire [68], Seoul Neuropsychological Screening Battery [42], Seoul-Instrumental Activities of Daily Living [42], Severe Mini Mental State Examination [47], Short Form Health Survey [37], Social Functioning in Dementia Scale [51], the Clock Drawing Test [41] and the Trail Making Test [41].

Within the second category (exercise/functional) eight outcome measures were identified across four studies and all outcome measures were reported in one paper each; Accelerometer [68], Actigraphs [30], Barthel Index [28], Functional Ability Tests [69], Inclinometer [68], Interview for Deterioration in Daily living in Dementia [28], Non-exercise equation to calculate cardio-respiratory fitness [68] and the Timed Up and Go-test [30]. The third category (qualitative) highlighted five outcome measures across twenty papers. Interviews were reported in 11 papers [23, 28, 29, 31, 32, 45, 51, 54, 56, 57, 60]. Observations were reported in eight papers [32, 38, 52, 53, 55, 57, 60, 65].Video recording in five papers [36, 38, 46, 50, 60]. Reflective discussions in two papers [38, 44] and photograph in one paper [46].The final category (activity) highlighted three outcome measures across three papers. Outcome measures identified were an activity observation form [36], verbal fluency [56] and a self-designed tool [71].

### Reported outcomes

A broad range of outcomes were identified within the papers reviewed. However, four papers identified no real difference [28, 41, 53, 67] and overall low to medium effect or maintenance post intervention was generally reported. These can be grouped under into four categories 1) increases in functioning, 2) social, 3) health and well-being and 4) enablement outcomes. Increases in functioning was reported in 16 papers across four elements: cognition [35, 37, 40, 42, 44, 45, 48, 51, 56] physical activity/ability, [30, 48, 63, 68–70] activities of daily living [42] and social skills [71].

Increases in social functioning was reported in 24 papers across eight elements. Connection/engaging with others was evident in 11 papers [32, 38, 43, 51, 54, 57, 60, 62, 65, 66, 71]. Increased communication was evident in seven papers. [36, 49, 50, 53, 56, 61, 72] Building relationships was evident in five papers [14, 31, 46, 54, 59]. Increased participation [29, 43, 48, 52] and increased social interaction [31, 32, 52, 53] were evident in four papers each. While increased enjoyment was evident in three papers [50, 57, 71] and increased group connection [57] and knowing the person [34] were evident in one paper each.

Health and well-being were reported in 17 papers across six elements and the main element was reduction in behaviours of concern [43, 47, 58, 63, 64, 66] Increased mood was evident in five papers [36, 42, 58, 59, 66].Three elements were all evident in three papers each: emotional well-being [38, 39, 70], increased well-being [33, 34, 63], quality of life [37, 61, 63]. The remaining element depression [35, 36] was evident in two papers.

Enablement was reported in seven papers across ten elements, two elements motivation [37, 57] and a sense of belonging [57, 60] were reported in two papers each. The remaining elements were all report in a single paper: a sense of accomplishment [57], increased agency [32], increased choice [52], collaborations [48], increased confidence [57], inclusion [59], a sense of freedom [32] and being self-conscious [57].

### Reported adaptations

Of the studies reviewed 19 reported on adaptations or adjustments made when implementing the psychosocial interventions. This provided important contextual factors around implementation. Five studies within the nature-based interventions grouping are relevant here. Ibsen and Eiksen [31] recounted how all the interviews took place at the farms so that participants could better remember or relate to the day care setting. While Noone and Jenkin [32] reported that over the course of the gardening project, the researcher developed a relationship with each participant which enabled participants to feel comfortable communicating their level of willingness to participate in a particular activity. Chang et al. [36] described the focus of each reminiscence session being on a particular life phase with objects relevant to that period introduced for discussion and incorporating the use of records and interviews about older people’s early lives and interests. Gregory [34] adjusted the timings of the intervention depending on factors such as the participant’s ability to concentrate and communicate and the intrusion of others into the space. The participants’ words were recorded and shown to them to emphasise how effectively they had been able to communicate their life stories and in read back sessions the poet read the participants’ poem aloud. Finished poems were sent to GP and kept in patient files. Lin et al. [37] revised the life review programme to compress it into a short format that was administered in 10 successive sessions over 2 weeks.

Within the memory/cognitive-based interventions grouping two papers are relevant. Jung et al. [42] reported that the main activity stage comprised activities for strengthening memory and management function and for increasing attention, concentration, and space-time, perception, concept formation and reasoning, composition, language, and computational abilities. Help was provided if required by the participant and initially, an easy level programme was gradually adjusted to appropriate and slightly difficult levels executed to develop person interest, sense of achievement and confidence. The programme differentiates from existing chair-based exercises by including bilaterally asymmetric activities that involves the left side of the body moving differently to the right side of the body as well as activities requiring mirroring a partner to rhythmic music that evokes memories and reminiscence. Kallio et al. [41] tailored training according to the participants’ cognitive abilities and it was implemented either in small groups of two to four persons, or individually when needed due to difficulties in concentration, or lack of a training pair. The difficulty level was tailored during the sessions, but it was not automatic as in computerized training.

Six papers within the social-based interventions grouping six papers reported adaptations or contextual factors that determined how the intervention was adjusted. Gjernes [52] described the staff member initiating discussions and serving as the engine that made the network social. Typically, the staff member used strategies to involve every person in the knitting activity. The knitters were supported to remember and participate in telling their own stories. The staff member was both a potential helper and a member of the social network. The staff could provide knowledge and skills when needed or could assist the knitters in their problem-solving efforts. Lancioni et al. [55] described how in the modified program version the computer presented photos and videos, providing encouragements to talk as well as attention and guidance. In another [58] individualised music playlists were developed by asking caregivers about the participant’s favourite music or by playing different songs for participants to see their reactions. During the study, some participants listened to their favourite songs repeatedly, others listened to a variety of songs. Distraction was minimised by closing the door and ensuring the participant’s physical comfort. Peeters et al. [60] reported how the researcher used the analogy of a house with different rooms, in which buttons represent doors to move between rooms to explain the navigation through the different screens of music collections management functionality. Hattford-Letchfield [46] reported how a comedy workshops did not work to an exact script but allowed scenarios to develop based on the main theme. Experiential drama techniques were used to work with the issues identified by the participants. A selection of photographs was made into a ‘scrapbook’ of the project as a whole that could be used as a reminiscence tool after the project had finished. Cheung et al. [56] adapted the Play Intervention for Dementia and integrated elements of cognitive stimulation in six identified mind–body functional domains and followed the principles of cognitive stimulation. During the sessions, participants could exercise their creativity in a cheerful and respectful environment, without anyone judging their (dis-)ability or without pre-set rules.

Within the animal-based interventions grouping two papers reported adaptations [61, 62]. Both papers described how sessions were designed to follow a protocol but could be individually tailored to each participant based on the care workers’ knowledge of the participant. Hence, none of the animal assisted activities were mandatory and the sessions also included naturally occurring activities between the participants as well as between each participant and the dog. Within the physical/sensory-based interventions grouping two papers reported adaptations. Karania^71^ describes how this programme differentiates from existing chair-based exercises by including concurrent bilaterally asymmetric activities that involves the left side of the body moving differently to the right side of the body, as well as activities requiring mirroring a partner to the sound of rhythmic music that evokes memories and reminiscence. Chang, et al. [69] described a bespoke exercise programme designed as a series of exercise training interventions aimed at maintaining activities of daily living abilities with adaptations made to the comedy workshops and dance activities. Aguinaga and Marquez [68] reported modifying their dance intervention where participants wore an orange Velcro bracelet on their right wrist and a green Velcro bracelet on their left wrist to help them distinguish between moves to the left and right. The programme was adapted as needed by revising the dance moves in ways that still challenged participants physically and cognitively but did not overwhelm them or put their safety at risk.

## Discussion

This scoping review illustrates the extent and range of psychosocial interventions being used in day care services. Forty-five papers were included in this review. Essentially, this review illustrated how there are many different types of psychosocial interventions and therefore these interventions belong to a ‘broad church’ in the sense of the myriad number and variety of psychosocial interventions being used. Crucially what they are all trying to do is to engage and support the person and enable them to connect and experience meaningful activities. The ability to connect is an important aspect of being ‘human’, as McCormack and McCance [72] have suggested that integral to being a person is being able to connect. This is especially so when a person is living with dementia as they experience challenges to their identity as people because of the effects of the dementia, therefore being able to connect with others, environment and self is key to being a person [72]. Personhood as a concept captures the uniqueness of personal identity and individuality of each person.

The psychosocial interventions described in this review illustrate how connections are made through and with the person through their physical body (sensory and motor), through time (immediate present and connecting with past), physical and psychological environment (space) and lastly through connecting with self and with others in a social world (relational). These connections of body, time, environment/ space and relationships resonate with the concept of lifeworld existentials of lived time (temporality), lived space (spatiality), lived body (corporeality), lived human relations (relationality) [73, 74]. The lived experiences of these connections are unique and individual to each person. Psychosocial interventions need to be provided from a person-centred holistic care perspective which supports the individual and their family [75, 76]. This will acknowledge the personhood and uniqueness of each individual and assist the person to self-actualise. There was strong evidence supporting the adaptation or modification of interventions as required based on individual client need in providing a person centred, flexible approach [31, 32, 34, 36, 37, 42, 46, 52, 55, 56, 58, 60–62, 69, 72]. Wyman and colleagues in a recent review reported key implementation factors that influence the impact of interventions, including, interventions tailored to stage of dementia, flexibility of delivery, participants engagement and carer supported [77].

This review illustrates the wide variety in the delivery and outcome aims of psychosocial interventions being provided, in so far as some are time limited, others open ended; some have very definite expected outcomes whereas others are less ‘directive’ or output driven. This may suggest that it is what is happening at this present time is the central or core focus of the intervention. People living with dementia can have cognitive and memory problems, (militating against remembering the psychosocial intervention once it is over) so it is the moment of connection that is important. The sense of connection was evident when creative therapy activities including dance, drama, music and movement were implemented and outcomes pointed to improved social skills and connection with those around them, among participants [71]. Meaningful connection with peers at the day care programme and connection with members of the community that alleviated some of the social and emotional isolation was reported when people living with dementia developed their life story project [38].

This review demonstrates how environment and space are important in helping people living with dementia connect. The use of the physical environment e.g. garden and green farms as psychosocial interventions [28–33] enable people living with dementia experience nature. Connecting with times past and past lives is evidenced by the use of psychosocial interventions such as reminiscence, life story and life review therapies [34–38] and as previously stated for some people, the present time is what matters as memory can be compromised.

The use of psychosocial interventions which enable connecting through bodily senses is facilitated by the use of psychosocial interventions which enable listening to and creating music [57–60], use of sight and hands to create art [51], tactile stimulation through multisensory therapy [65–67], animal therapy [61–64], robotic therapy [47] and exercise to engage the body to move [68–71]. Whilst not the focus of this review, Öhman et al. [78] emphasise the positive effect of physical exercise on cognition, this connection between mind and body may warrant further exploration within the context of psychosocial interventions. The ability to connect through relationships with others and with self are evidenced by the use of emotional/cognitive based psychosocial interventions which facilitate engagement of the mind [39–44] as well as the social interventions of music, art, multisensory, robotics, animals therapy as outline above as well as engagement in activities based psychosocial interventions [14, 45, 46, 48–50, 52–56]. This shows how psychosocial interventions can help connect the mind, body, temporal and spatial aspects of the person living with dementia.

## Strengths and weaknesses of the review

To our knowledge, this is the first scoping review conducted to identify the psychosocial interventions used in day care service for people living with dementia. This review was conducted in compliance with a recognised review methodology framework [21–25]. The main strength of this review is its inclusivity through the broad definition of day care and psychosocial interventions and that the authors searched widely for evidence in diverse fields. The review was limited to 2011–2023 which ensured currency of the studies included. Despite searching eleven databases there are rather fewer studies on exercise and activity interventions than might be expected. Including these search terms or including non-English language studies may have yielded more studies with a focus on exercise and activity interventions. As the focus of a scoping review is on identifying, mapping and charting; critical appraisal and risk of bias assessments were not completed but it is acknowledged they are not a requirement for scoping reviews [79]. A further limitation of this review is the fact that the focus is on mapping and describing the evidence and the authors do not identify or discuss the efficacy of the reviewed interventions. Thereby the findings do not support the definitive evidence of the efficacy of these interventions however, they show potential and require further investigation.

## Conclusion

This review aimed to identify the psychosocial interventions provided in day care services and it has illustrated the wide variety in the types, range and facilitation of these interventions. The review also highlights the need for and importance of supportive interventions that are person-centred and offer a sense of connection for people living with dementia. This review highlights the potential benefits of these interventions. However, findings must be considered in the context that many were provided as brief interventions with little evidence of longituidinal studies and further research is required given the complex and diverse range of interventions used in day care services for older people living with dementia and without, repeat measures post interventions to identify if short term benefits were sustained. Further research is required given the complex and diverse range of interventions identified. Nonetheless, results will be of interest to practitioners planning to implement or evaluate psychosocial interventions used in day care services for older people living with dementia.

## Supporting information

S1 File Search strategy. (DOCX)

S2 File Data extraction table. (DOCX)
