# Supplementary material for: A scoping review on the psychosocial interventions used in day care service for people living with dementia
Source: PLoS One. 2023 Dec 11;18(12):e0295507. doi: 10.1371/journal.pone.0295507 (PMC10712883; doi:10.1371/journal.pone.0295507)
Supplement: S1 File — (DOCX) [file pone.0295507.s001.docx]

**Supplementary File 1: Search Strategy**

**Table S1: Search strategy of CINAHL Complete database - Total: 1638 papers, undertaken 3^rd^ February 2023**

| **Search** | **Search terms/modes - Boolean/Phrase** |
| --- | --- |
| S11 | S5 AND S6 AND S10 |
| S10 | S7 OR S8 OR S9 |
| S9 | TX “cognitive therapy” or “cognitive behavioral therapy” or cbt or “cognitive behavioural therapy” or “brain training” |
| S8 | TX counsel* or therap* or psychotherap* |
| S7 | TX Psychosocial or psychological or social |
| S6 | TX day N3 (care or cent* or service* or facilit* or program*) |
| S5 | S1 OR S2 OR S3 OR S4 |
| S4 | TI ("creutzfeldt-jakob disease" or "creutzfeldt-jakob syndrome" or cjd or "huntington* disease" or "posterior cortical atrophy" or "Korsakoff Syndrome" or "normal pressure hydrocephalus" ) OR AB ( creutzfeldt-jakob disease or "creutzfeldt-jakob syndrome" or cjd or "huntington* disease" or "posterior cortical atrophy" or "Korsakoff Syndrome" or "normal pressure hydrocephalus" ) |
| S3 | TI ("neurocognitive disorder*" or "lewy body dementia" or parkinson* or "parkinson* disease" or "frontotemporal dementia" ) OR AB ( "neurocognitive disorder*" or "lewy body dementia" or parkinson* or "parkinson* disease" or "frontotemporal dementia" ) |
| S2 | TI (dementia or alzheimer* or "alzheimers disease" or "vascular dementia") OR AB (dementia or alzheimer* or "alzheimers disease" or "vascular dementia") |
| S1 | MH "Dementia+") OR (MH "Frontotemporal Dementia+") OR (MM "Alzheimer's Disease") OR (MM "Parkinson Disease") OR (MM "Creutzfeldt-Jakob Syndrome") OR (MM "Huntington's Disease") OR (MM "Korsakoff Syndrome") OR (MM "Hydrocephalus, Normal Pressure") |
