# Supplementary material for: A scoping review on the psychosocial interventions used in day care service for people living with dementia
Source: PLoS One. 2023 Dec 11;18(12):e0295507. doi: 10.1371/journal.pone.0295507 (PMC10712883; doi:10.1371/journal.pone.0295507)
Supplement: S2 File — (DOCX) [file pone.0295507.s002.docx]

**Supplementary file 2: Data Extraction Table**

| **Author, Year,**  **Country** | **Aim** | **Research Design** | **Type of psychosocial interventions** | **Use and facilitation of psychosocial interventions** | **Evaluation methods used for psychosocial interventions** | **Reported psychosocial interventions outcomes** | **Reported adaptations to psychosocial interventions** |
| --- | --- | --- | --- | --- | --- | --- | --- |
| Aguinaga & Marquez  2019  USA | To determine whether a Latin dance program with sedentary behaviours information would have an impact on physical activity, cardiorespiratory fitness (CRF), and sedentary behaviours among older Latinos attending an adult day centre (ADC). | Design: Pilot RCT  Sample: N= 21  Data Collection: Participants were randomized into a dance or wait-list control group. Participants wore an accelerometer and inclinometer and completed a sedentary behaviours questionnaire, and a non-exercise equation was used to calculate CRF.  Ethics Statement Provided. | A modified dance programme called BAILAMOS © included a 4-month, twice-weekly program. Every dance session was 1 hr in length & included four dance styles: Merengue, Cha Cha Cha, Bachata, and Salsa. | Monthly discussion sessions are also held in which a research assistant focused on increasing knowledge, outcome expectations, social support, and self-efficacy to increase lifestyle physical activity among participants.  Research staff member were present at all dance sessions to set up the room and observe the class. | Participants wore an accelerometer and inclinometer and completed a sedentary behaviour questionnaire, and a non-exercise equation was used to calculate cardio-respiratory fitness (CRF). | Findings indicate small to medium effect sizes in the desired direction during midpoint of the intervention for physical activity, sedentary behaviour–related outcomes, CRF, and self-reported sedentary behaviour in the dance group; however, dance participants did not maintain that trajectory for the remaining 2 months of the intervention. | Several changes were made to this PSI (BAILAMOS©). Participants wore an orange Velcro bracelet on their right wrist and a green Velcro bracelet on their left wrist to help them distinguish between moves to the left and right. The program was adapted as needed, revising the dance moves in ways that still challenged participants physically and cognitively but did not overwhelm them or put their safety at risk. |
| Baker & Stretton-Smith  2017  Australia | To explore how four people living with mild to moderate dementia described their experiences of a pilot community-based group therapeutic song writing (TSW) program. | Design: Open, exploratory research, an interpretivist research design, and interpretative phenomenological analysis (IPA).  Sample: N=4 participants with dementia (purposive selection) and N= 3 support staff.  Data Collection: Interviews  Ethics Statement Provided. | Ten one-hour song writing sessions were facilitated once a week by two music therapists, one author and another unaffiliated registered music therapist. | Sessions took place in a large activities room at the day centre, and group size ranged from six to ten people with dementia (PWD), as well as up to three staff members. Leadership changed fluidly as needed throughout each session, with one music therapist leading the song writing experience while the second music therapist supported the leader. | One-on-one semi-structured interviews allowing for sensitivity, reflexivity, and flexibility were conducted of 20/25-minute duration. Support staff, who were co-participants in the song writing group, also reported their observations via interview. | TSW was emphasized as a positive, enjoyable, and rewarding experience. Both PWD and staff described TSW as: (a) motivating participation; (b) enhancing confidence to actively engage; (c) highlighting ability and leading to feelings of accomplishment; (d) stimulating engagement in creative, cognitive, language, and learning processes. Participant collaboration within song writing was described as enhancing feelings of connection, belonging, and group cohesion, while sometimes also highlighting challenges and leading to feelings of self-consciousness. | Not Reported. |
| Chang, Hui Chen,  & Chien, Hui-Wen  2018  Taiwan | To explore as a pilot study the effects of group reminiscence therapy (GRT) upon depression of people living with dementia within dementia-specific day care centres. | Design: Quasi-experimental design  Sample: N= 21 from two dementia-specific day care centres  Data Collection: Pre-test-post-test instruments counterbalanced with a qualitative grounded theory video analysis of facial expression and behaviours used to measure the effects of GRT.  Ethics Statement Provided. | Small groups of four to five participants had a series of six group sessions of reminiscence therapy, one hour each, over a six-week period. The topics for the sessions were generated from Life Story interviews of 1–2 hours in length held once a week for a month prior to the commencement of the study. | Individual RT sessions once weekly x six weeks x 1 hour, using general reminiscence approach identical for individual and group sessions.  Primary researcher, who specialized in dementia care and reminiscence therapy, conducted the intervention and led group activities.  Two research assistants served as group co-leaders who observed interactions among group members, encourage group discussion, and protect participants from losing emotional control during activities and record participants’ emotional and behavioural changes.  Two staff familiar to the participants from the day care centre assisted with each section to facilitate discussion and help participants when needed. | Instruments included 1) mini-mental state examination MMSE, 2) Cornell Scale for Depression in Dementia CSDD, 3) activity observation form 4) video recording.  Assessments were carried out immediately before and after the intervention. | The findings highlighted improvements in depression, communication, and positive mood after group reminiscence intervention.  Results showed that participants scored higher on average during the “Chinese New Year” and “marriage” reminiscence activities compared to the other activities, revealing the subject matters’ extraordinary significance, and concluded by supporting the proposition that undertaking a cultural focus reminiscence therapy may produce significant psychosocial improvements for a person with dementia. | Focus of RT sessions on a particular life phase in each session and introduced objects for discussion relevant to that period. Used records and interviewing each participant, as well as their caregivers and their family members about elders’ early lives and interests. |
| Chang, Su-Hsien, *et al*.  2011  Taiwan | To examine the effectiveness of an exercise programme for elders with dementia on the maintenance of their activities of daily living such as feeding, dressing, grooming, washing and toileting. | Design: Single study group, repeated measure research design  Sample: N= 26 dementia elders were recruited from a day-care centre  Data Collection: The Refined ADL Assessment Scale (RADL) and functional ability tests.  Ethics Statement Provided. | Caregivers of the day-care centre provided all subjects an exercise programme. The exercise programme consisted of stretching and walking five times per week, and leg-weight bearing at least three times per week for 20–30 min each. The data were obtained three times (baseline, 2 months post intervention and 4 months post intervention). | The exercise programme consisted of stretching and walking exercises five times per week, and leg-weight bearing three times per week for 20–30 min each. In the morning, subjects were encouraged to walk 20–30 min while listening to their familiar music. The walking exercise was preceded and followed by 5 min of stretching exercise to warm up and cool down. After the walking programme and weight training programme, each subject was given a ‘star’ for his or her achievement. The ‘star’ was stamped on a blackboard at the nursing station and could be viewed by other elders. Furthermore, elders who regularly performed exercise were given positive feedback and encouragement. | The RADL was used as it was designed for elders with Alzheimer’s disease and related disorders. Functional ability tests included, one leg standing, functional reach, thirty second chair rise and get up and go. | Results showed slight changes in the scores of one-leg-standing, 30s chair rise, functional reach and get up and go test but were not statistically significant. Results also indicated that scores in the performance of activities of daily living were significantly higher than at baseline and at 4 months post intervention. | Bespoke exercise programme designed and operationally defined as a series of exercise training interventions aimed at maintaining ADL abilities for dementia elders. |
| Cheung *et al*.  2019  Hong Kong, China | To investigate the feasibility and the preliminary efficacy of a Cognitive Stimulating Play (CoS-Play) intervention on cognitive functions. | Design: RCT  Sample: 30 participants recruited from 2 centres (CoS-Play group: n=18; control group n= 12) and 2 staff members working in the intervention site were interviewed to evaluate the feasibility and preliminary efficacy of the intervention.  Data Collection:  1. Montreal Cognitive Assessment  2. Fuld Object Memory Evaluation  3. Modified Verbal Fluency Test and  4. Interviews with staff members  Ethics Statement Provided. | Eight weekly group-based sessions of the CoS-Play, each 45 to 60 minutes in length.  Activities comprising 6 elements: visuospatial and fine motor activities (e.g., making handicrafts), kinaesthetic and gross motor activities (e.g., batting a balloon), language and verbal activities (e.g., telling stories), executive function activities (e.g., card games), auditory music and rhythmic activities (e.g., playing percussive musical instruments), and social interaction. | Control and intervention group.  The control group took part in social activities (reading newspapers, watching television) in a similar pattern as the intervention group. | Effects on cognitive functions were measured with statistical analysis using SPSS.  The mean memory storage and retrieval functions were 5.92 and 4.12 points higher than that for the control group, respectively, which contributed moderate to large effects. There was no significant difference between groups in global cognition measured using the Montreal Cognitive Assessment and verbal fluency.  Deductive content analysis to analyze interviews in relation to the feasibility of the intervention, around acceptability, integration, practicality, and effects. | Feasibility and acceptability of using the intervention was measured -several practical issues that might affect its implementation, the CoS-Play can feasibly be implemented, and with positive effects on the participants. The intervention was acceptable to both the PWD and the staff working in the community centres. | The CoS-Play (an adaptation of original *Play Intervention for Dementia*) integrates elements of cognitive stimulation in 6 identified mind–body functional domains and follows the principles of cognitive stimulation. During the sessions, participants can exercise their creativity in a cheerful and respectful environment, without anyone judging their (dis-)ability or with any pre-set rules. |
| Ching-Teng  2019  China | To test the effectiveness of board game activities on improving the cognitive function of older adults in adult day care centres. | Design: Quasi-experimental  Sample: N= 82 (purposive sample)  Control Group N=41  Intervention Group N=41  Data Collection: structured questionnaires and data analysis using SPSS 22  Ethics Statement Provided. | The board game sessions were conducted for 90 min each for a total of 12 weeks, led by a recreational therapist. | Led by an experienced recreational therapist. Intervention conducted in a small group setting, subjects randomly assigned to six teams of five or six people each. Before the activity, the physical and mental states of participants were examined, and the social worker ensured the safety of the participants during the activities. During an expert panel discussion involving a psychologist, nursing professor, recreational therapist and social worker all experienced in working with older adults, various board games were selected for the sessions. | Structured questionnaire comprising demographic questions, a Geriatric Depression Scale (GDS-SF), and a Montreal Cognitive Assessment Scale, test duration was 15–20 min. Cognitive assessments given to the participants 1 week before and 1 week after the intervention. Cognitive function was collected at baseline during a face-to-face interview conducted by a psychologist. | The board game programs showed promising effects in the cognitive function of older adults living in adult day care centres. A possible beneficial effect of board game playing on the risk of dementia could be mediated by a less cognitive decline in older adults. Board game activities may benefit the cognitive function of older adults. Incorporating board game activities into social work care may help develop long-term care into a more diverse, unique and innovative direction. | Not reported. |
| Dabelko-Schoeny *et al*.  2014  USA | To determine the feasibility and effectiveness of using guided interactions with horses as a nonpharmaceutical intervention to improve the physiological and behavioural states of persons with dementia. | Design: Randomized pre-test-post-test crossover design  Sample: N= 16  Data Collection:   1. Behavioural Observation Recording 2. Salivary Cortisol 3. Modified Nursing Home Behaviour Problem Scale (NHBPS)   Researchers compared participants receiving the equine-assisted intervention with participants receiving treatment as usual. | The equine-assisted therapy intervention program occurred once a week on the same day for four weeks and consisted of three specific intervention activities.  Each activity was a 15-minute hands-on experience with the horse followed by 5 minutes of rotation to the next activity. | All interventions were groundwork only. Activities included, 1. Grooming, 2. Observation of horse-to-horse interactions as well as allowing the participant to seek interaction with the horse 3. Leading  the horse around the arena and placing and removing a halter 4. Painting with symbols, 5. Washing and reward feeding horses.  Activity groups had two or three members who remained in the same group for the duration of the study. One adult day services staff person per activity group was available to assist participants with wheelchair mobility, standing, or walking. Additionally, each participant was aided by one equine assisted-learning staff to ensure safe engagement with the horse. | Behavioural Observation Recording Two trained research observers made recordings of participant behaviours and affect every 30 seconds during the 15-min. intervention at two of the stations: grooming and painting. Recorded the participants’ affect by using a modified PCG Affect Rating Scale including recording expressions of pleasure, interest, neutral, anxiety/fear, anger, or sadness. Pre- and post- intervention salivary cortisol levels were collected as an indicator of physiological stress. Modified Nursing Home Behaviour Problem Scale (NHBPS)- On a weekly basis, day centre staff completed a modified version of the NHBPS at pre-test, immediately following the intervention, and at post-test. | Findings suggest engagement in equine-assisted therapy may reduce problematic behaviours that can be exhibited by individuals with AD attending ADS centres. Equine-assisted therapy is a feasible, non-pharmacological intervention to reduce problematic behaviours among people with AD, resulting in easier caregiving for formal and informal carers. This therapy may provide a tailored intervention that can positively impact the quality of life of the person living with AD and their caregivers. | Not reported. |
| De Bruin *et al*.  2021  The Netherlands | 1) to understand the motivation of people with dementia (PWD) and their family carers to choose nature-based adult day services (ADSs) in urban areas  2) increase understanding of the value of nature based ADSs in urban areas in terms of the health and wellbeing of PWD and their family carers from different perspectives. | Design: Qualitative  Sample: N= 39  Data Collection: Semi-structured interviews were conducted with people with dementia, their family carers and providers of nature based ADSs in urban areas.  Ethics statement provided | Broad description of nature based ADSs was provided, non-specific. | Not specific to one site as participants were from Nature based ADSs across The Netherlands. | Conducted 39 interviews: 21 with participants and 18 with family carers.  Framework analysis method was employed to analyse the data. | Nature-based ADSs in urban areas have a wide range of benefits that might affect the health and wellbeing of people with dementia and their family carers. | Not reported. |
| De Bruin *et al*.  2012  The Netherlands | To compare longitudinal change in functional performance in community-dwelling older people with dementia who attend day care at green care farms (GCFs) or at regular day care facilities (RDCFs) | Design: Observational cohort study  Sample: primary caregivers of 47 subjects from GCFs and 41 subjects from RDCFs rated the subjects’ functional performance three times for one year.  Data Collection: Interview  Ethics Statement Provided. | Attendance at GCFs – no further PSI described. | Attendance at GCFs – no further PSI described versus attending RDCFs. | Cognitive functioning of the subjects was assessed by the Mini State Examination (MMSE). Functional performance was rated by the primary caregivers by means of the Barthel Index (BI) and the Interview for Deterioration in Daily living in Dementia (IDDD). Primary caregivers of the subjects were interviewed three times at their homes with six-month intervals (at study entry, at 6&12-month follow-up). Except for one subject, primary caregiver were family caregivers such as spouses or children. | Generally, no significant change over time in functional performance, the number of diseases and the number of medications was observed, and no differences in these rates of change were found between subjects from both day care settings. This study suggests that GCFs are not more effective in maintaining functional performance or slowing down its decline in community-dwelling older people with dementia than RDCF. | Not reported. |
| De Bruin *et al*.  2015  The Netherlands | To explore the value of day services at green care farms (GCFs) in terms of social participation for people with dementia (PwD) living at home | Design: Qualitative descriptive.  Sample: N=51 PwD and their family caregivers.  Data Collection: semi structured interviews.  Ethics Statement Provided. | Not described. | Standard participation in GCFs | Semi-structured interviews with participants and their family caregivers. Three groups: (a) PwD who attended day services at a GCF (GCF group), (b) PwD who were on a waiting list (WL) for day services at a GCF (WL group), and (c) PwD who attended day services in a more traditional ADS setting, in this study, operationalized as a facility affiliated with a residential home (i.e., regular day care facility; RDCF group). | GCFs are valuable in terms of social participation for a particular group of people with dementia. | Not reported. |
| Finnanger Garshol *et al.*  2020  Norway | To investigate potential of FBDC to promote physical activity for PWD.  Study 1: To gain knowledge of experiences and health benefits of attending FBDC among PWD and their next of kin.  Study 2: To if attending day-care centers would have an effect on factors related to risk of fall, accidents, balance and QoL. | Analysis of data collected in two separate studies.  Study 1: Prospective Protocol longitudinal study. No age inclusion criteria.  Study 2: 65 or older, PWD  attending FBDC services (n = 29) and people with dementia attending regular day care services (n = 107). | Actigraphy data was used to assess levels of physical activity in each group and to compare the two groups. | Both studies used a test of physical functioning to assess functional mobility and an actigraph to measure levels of physical activity.  Study 1: data collected at the second data collection point 6 months after baseline – Study 2: similar data as in Study 1 collected from participants attending regular day care services for people with dementia. | Used Actigraphs (Actisleep+, Actigraph, Pensacola, US) to measure the level of physical activity.  Participants wore the actigraph on the left wrist continuously for 7 days, these days included both days while at the day care services and days while not at the day care services.  Clinical Dementia Rating (CDR) scale to assess level of dementia Timed Up and Go-test (TUG). | For the group attending FBDC services, days at the service, were significantly associated with less time spent in sedentary activity (p = 0.012) and more time spent in light (p < 0.001) and moderate activity (p = 0.032), and in taking more steps (p = 0.005) compared to days not at the service. | Not reported. |
| Gjernes,  2017  Norway | To study social dimensions of dementia: how people with dementia interact and solve problems while participating in social activities with a focus on knitting as the main activity. | Design  Ethnography.  Sample  Day Care Centre with 40 service users where ersona. 20 attend each day. Participants: elderly people with various types and degrees of dementia, people with minor intellectual disabilities, people with physical disabilities, and a few elderly people who were lonely. Staff members (N=3).  Data Collection  Observation, field notes, reflections, observations, conversation, interviews.  Data Analysis  Thematic analysis.  Ethics Statement Provided. | Social Activities main focus Knitting  (meals, bingo, exercises, reading periods, knitting, dancing; clearing tables after meals, reading, crossword puzzles, games, talking). | Over 8 weeks, researchers spent 4-6 hours daily at the day center, participating in organized activities. Knitter participants varied from day to day, (6-9 women). Some attended the day center 5 days a week, whereas others came 2/3 days a week.  The knitting activity and group were organized by staff members at the center. The aim of the group was to involve group members in a shared activity helping them create a social group. Knitted elements were processed into a complete knitted product by staff and sold. Although they knitted, the women talked about knitting and various topics related to everyday life, and the topics of discussion were initiated by the knitters or by a staff member. | Researcher commentary: Significance of activity, collaboration, and social togetherness was essential for the maintenance of people’s body and social functions. In addition, the knitters enjoyed themselves and looked forward to coming to the day center, meeting friends, and having a structured, daily routine. Thus, participating in day center activities and the knitting promoted well-being, and also meaning during the fourth age. | Themes  The Day center  *Activities in the Parlor*  Social Interaction and Participation  *Social Communication, Participation, and Scaffolding*  C*ollaborative Problem Solving.*  The social participation and collaboration during problem solving among the knitters and between the knitters and the staff member supported the cognitive capacities of the knitters and reduced the social and practical consequences of individual cognitive limitations. Knitters’ cognitive capacity emerged through practice, as did their ability to accomplish practical and theoretical tasks. | Staff members served as the engine that made the network social using strategies to involve every person. The knitters received support to remember and participate in telling their own stories. Staff members were both potential helper’s and members of the social network providing knowledge and skills when needed or assisting knitters in their problem-solving efforts. |
| Gregory,  2011  UK | To explore experiences of care staff of the impact of a *Try to Remember* poetry intervention at 3 residential care homes and one day centre (Arbour House, AH) in England. | Design  Qualitive Evaluation IPA.  Sample  One residential care home (Elm View) and one day care centre (Arbour House).  Six care staff (1 male, 5 female, 41-63 yrs)  who participated in a poetry intervention with individuals with diagnoses of dementia ranged from mild/moderate (7 cases) to severe (14 cases).  Data Collection  Individual in-depth, semi-structured interviews with care staff.  Data Analysis  IPA.  Ethics Statement Provided. | *Try to Remember*  Intervention consisted of three core components: reminiscence sessions with service users; readback sessions with service users, family members, care staff and other health care professionals, such as local GPs; and skills training workshops with care staff. | Twenty-one PWD and 11 care staff involved in the project delivered over four weeks: Reminiscence sessions averaged 40 mins. Clients typically seen x2/3 times. Readback sessions lasted around 50 min. Two three-hour workshops were held at each site: listening skills; non-verbal communication; writing skills; and boredom, anxiety and isolation amongst clients. The poet recorded individuals’ words and showed same to the client to emphasize how effectively they had been able to communicate their life stories to her. In readback sessions the poet read the clients poem aloud. Finished poems were sent to GP and kept in patient files. | Not stated. | 6 themes: exploring and preserving memories, communicating with service users, humanizing dementia sufferers, co-authorship of poems, continuity and the broader care context. Staff valued intervention as having a positive impact on the well-being of clients/ families. Reminiscence based poetry can help restore personhood to dementia sufferers by providing carers with a fuller understanding of clients’ past and present lives, enabling and encouraging carers to communicate more effectively with those in their care, allowing clients to engage in productive activities and facilitate meaningful social interactions. | Session length varied depending on factors such as the participant’s ability to concentrate and communicate, and the intrusion of others into the space. |
| Hattford-Letchfield,  2012  UK | To use comedy as a medium to explore alternative ways of learning with older people and confronting issues or stereotypes around ageing and well-being in later life using arts-based approaches. | Design  Experiential Evaluation.  Sample  A day centre for older people with dementia. 12 older service users, 4 family carers, 8 staff and 2 comedy trainers, supported by a project leader and a person responsible for filming and technical issues.  Data Collection  6 hours of digital recording and voluntary recording of planning and debriefing meetings with staff, family carers and the project team after each workshop.  Data Analysis  Not stated.  Ethics Statement Provided. | Comedy Activities Workshops. | Using improvisatory activities and comedy, to engage older people in reflecting on aspects of their care environment, four workshops lasting 2/3 hours each ran weekly for 4 weeks; held at the same day and time each week. Workshop 1: Grangers and staff receive the news that the Queen is planning to visit their day centre and much excitement and planning begins. Workshop 2: the day centre makes practical preparation for the Queen’s visit. Workshop 3: Grangers prepare their entertainment for the Queen’s visit. Workshop 4: Her Majesty the Queen visits the Grange. | All of the workshops were continuously filmed and photographed. Footage from the former was edited to make the final 30-minute mockumentary. The photographs were printed after workshop two and used during the two subsequent workshops as an ‘aide memoir’, and as a reflective tool for promoting discussions between service users, carers, staff and the project team about ‘how it was going’ between each weekly workshop. | Themes from the filming and interview (being in the moment, Using comedy to ‘challenge’ stereotypes, The benefits of the project in building relationships.  The main output resulted in a 30 minute ‘mockumentary’ of the ‘Her Majesty the Queen’ visiting the day centre, in the form of a digital reusable learning object to be used by social work and mental health professionals. At the end of the project, all the photographs were printed and distributed to individual participants for their ‘life-story’ work. | The workshops did not work to an exact script but allowed scenarios to develop based on the main theme. Experiential drama techniques were used to work with the issues that Grangers came up with themselves. A selection of photographs were made into a ‘scrapbook’ of the project as a whole that could be used as a reminiscence tool after the project had finished. |
| Ibsen and Eriksen  2021  Norway | To investigate how people with dementia describe attending experience attending FDC services in Norway. | Design  Qualitative descriptive  Sample  10 people diagnosed with dementia recruited from five FDCs in Norway (age range 60-90)  Data Collection  Individual semi-structured  Data Analysis  Content Analysis  Ethics Statement Provided. | Farm-based day care service | Between May and November 2018, in FDCs the structure of the day was similar at the five participating farms and included breakfast, work session, coffee break, walk or other activities and then dinner before leaving | When describing experiences all participants were asked if there was something they did not like at the FDC or if something should be different. | The overall theme: ‘attending day care at a farm makes me feel like a real participant’ included three main categories: (1) social relations, (2) being occupied at the farm and (3) individually tailored service. PWD described how they played an active part in their relationships with others and in the work and help they provided at the farm. The farm context was described as a natural setting for outdoor activities, and the work tasks were characterized as authentic farm work that needed to be done. The participants were allowed to use their competency at different tasks at the farm, and they had the opportunity to have an influence on their stay at the day care. | All of the interviews took place at the farm, so the participants could better remember or relate to the day care setting |
| Ihara *et al.,*  2018  USA | To examine the effects of a person-centered music listening intervention on mood, agitation and social engagement for ADHC participants living with dementia. | Design  Quasi-experimental.  Sample  5 community-based ADHCs. Participants (n= 51), with a dementia diagnosis  33 – intervention condition 23 – comparison condition.  Data collection  Data for standardized instruments (Mood and Agitation) collected pre-post- 6 weeks post intervention for both conditions at the same time. Behavioral observational data collected each time the intervention was administered, twice weekly for 6 weeks for the IG.  Data Analysis  Descriptive statistics.  Ethics Statement Provided. | *Music and Memory* an individualized music listening system/program. | Individuals were given an iPod programmed with their personalized playlist. During the study, some participants listened to their favorite songs repeatedly, others listened to a variety of songs.  To assess changes in mood and agitation, Intervention group listened to their personalized music, comparison group participated in daily planned activities. Some individuals interacted with one another and/or researchers while listening to songs. | Mood and agitation assessed three times – pre-post-and 6 weeks post-intervention.  Depression – CSDD. Agitation – CMAI. Both completed by family caregivers. Within-person differences examined using the Wilcoxon signed rank test, and between-group differences examined using the Mann–Whitney U-test.  In-person and video-recorded observations of participant behavior were used to analyze changes before, during and after intervention across 4 domains: mood, agitation, connecting to music and engaging socially. | 2 participants dropped out, resulting in 31 – intervention condition and 20 -comparison condition.  Although standardized instruments did not yield statistically significant results, the behavioral observations showed a positive change in mood and a decrease in agitation. From pre- to post-intervention, there were statistically significant increases in joy, eye contact, eye movement, being engaged and talkativeness, and a decrease in sleeping and moving or dancing. Study findings imply that personalized music has potential as a non-pharmacological intervention, particularly when the music choices are connected to positive memories for the individual living with dementia. | Individualized music playlists were developed by asking caregivers about the participant’s favorite music or by playing different songs for participants to see their reactions. To minimize distraction, the door was  closed, and  participant physical comfort ensured. |
| Jaana,  2020  Canada | To review the feasibility and contribution of an adult day care program (ADP) model to community services for  people living with dementia and their families. | Design  Case Evaluation.  Sample  Site for health professional practicum. Modified Apartment type environment in a Canadian College. Usually 30 seniors with dementia enrolled (10 per-day) three days a week and also supported at home by 29 caregivers.  Data Collection  A triangulation of data sources including existing documents, seniors’ records, and caregivers’ survey was distributed to a sample of 29 caregivers one year after the programme became operational.  Data Analysis  Descriptive data analysis conducted on SPSS.  Ethics Statement Provided. | Adult Day Program (ADP)  A coordinator was assigned to each senior client/caregiver for programme duration. The coordinator conducted weekly follow-ups with  the caregiver, provided support made referrals on a need basis when issues arose. | The ADP operated 50 weeks per year, 3 days a week. Individuals participated in two consecutive morning sessions, involving fitness fun and cognitive activity. Two afternoon sessions included; a) a physical activity session and a theme presentation; b) a media moment and walk around the block activity. The design of the apartment that hosted the ADP included diverse section areas (e.g. kitchen, bed, sitting area, etc.) offering seniors a comfortable and safe environment with flexibility to rest and relax as needed. Individualized care plans and assessment data are also shared with the seniors, caregivers, and health care providers. | Caregivers’ survey  Part 1: questions on the seniors’ condition in relation to behavioral problems and their level of activities using a scale adapted from Teri et al. (1992); 5 ADLs and 5 IADLs were assessed. Part 2: questions that assessed caregivers worry and strain, support services and satisfaction with the program.  Data on the utilization  of services and number of days spent in the program were extracted from existing records. | A total of 21 caregivers completed the survey, representing a 72.4% response rate. Seniors’ characteristics, behavioral problems, and level of activities fostered collaboration between a community resource center and an academic institution, which increased access to services for seniors and their caregivers and allowed students to get hands-on training in a real-life setting with seniors. Most importantly, it allowed seniors living with dementia to benefit from a carefully designed, safe and flexible environment, which was developed to ensure their participation in independent cognitive and fitness activities. Caregivers’ satisfaction with the ADP was very high. | Not Reported. |
| Jung et al.,  2020  Korea | To investigate the effect of integrated cognitive intervention therapy on cognition, and activity of daily life (ADL), and mood in patients with mild to moderate AD. | Design Pre-Post.  Sample  59 out of 93 patients with mild to moderate AD who met the NINCDS-ADRDS criteria of probable AD among those who registered at the Centenarian’s Good Memory Program from September 2014 to August 2019.  Data Collection (completion of outcome measures).  Data Analysis  Statistical analysis using PASW Statistics 23.  Ethics Statement Provided. | Integrated cognitive intervention therapy programme comprising cognitive training, music therapy, and art therapy.  The main activity stage comprised activities for strengthening memory and management function and for increasing attention, concentration, and space-time, perception, concept formation and reasoning, composition, language, and computational abilities | 32-hour therapy (2 hours×16 sessions). The mean number of sessions that the participants received was 14.7±1.3. Cognitive training comprised of 16 sessions of 60 minutes each conducted over 8 weeks (twice weekly). The training consisted of 5 stages: introductory activity, brain health lifestyle education, activity briefing, main activity, and finishing activity. The music therapy program was comprised one 60-minute session per week that was conducted for a total of 8 weeks. | K Korean version of a Mini-Mental Status Examination (K-MMSE), Korean Dementia Screening Questionnaire-Cognition (KDSQ-C), Geriatric Depression Scale (GDS), Beck Anxiety Inventory (BAI), and Seoul-Instrumental Activities of Daily Living (S-IADL) Seoul Neuropsychological Screening Battery (SNSB). | K-MMSE improved from 18.7±4.5 before therapy to 19.7±5 after therapy (p<0.001), the comparison between pre and post therapy scores of K-MMSE, KDSQ-C, S-IADL, GDS, and BAI indicated a significant improvement in cognitive functions, ADL, and patient mood after the implementation of the integrated cognitive intervention program.  The study demonstrated the possibility that the well-programmed integrated cognitive intervention therapy at the Centenarian’s Good Memory School significantly improves cognitive function, ADL, and mood in patients with mild to moderate AD. | Help was provided if required by the participant. Initially, an easy level program was gradually increased to appropriate and slightly difficult levels executed to develop person interest, sense of achievement and confidence.  The inclusion of bilaterally asymmetric activities and activities requiring mirroring a partner to rhythmic music that evoked memories and reminiscence was how this programme differentiated from existing chair-based exercises. |
| Karania,  2017  UK | To evaluate the impact of a bilaterally asymmetric gymnastics-based exercise programme on older people participating in a care home and day centre setting. | Design  Small-scale pilot study. Qualitative evaluation.  Sample  2 care homes and one day care centre in England.  Number attending sessions typically 12 – 16. All participants had a diagnosis of dementia.  Data collection  Interviews  Data analysis  Thematic analysis  Ethics Statement Provided | The programme content designed by the British Gymnastics Foundation, in collaboration with the person delivering the sessions, was made to be interactive with lots of humour and contact through partner activities; and it cognitively stimulating with the use of bilaterally asymmetric activities and memory evoking music. | This program involved active music activities split into three phases: introductory activity, main activity, and finishing activity. The art therapy program comprised one 60-minute session per week that was conducted for the duration of 8 weeks.  The programme was delivered by 3 organisations. Each session lasting 1 hour was delivered by the same person. One or two residents had a family member attend. Care home activity lead attended and occasionally other staff members. The delivery of each session was structured around eight core activity themes: meeting and greeting; gentle warm-up exercises; facial expressions and arms crossing the mid-body line; bilaterally asymmetrical movement patterns; paper, scissors, stone game; partner working; cognitive stimulation activity and relax and wind down exercise. | Not stated. | Themes: Posture and movement, Emotions and memory, Social engagement and quality of life, Older people supported and frequency of programme.  Older people participating in the programme showed a demonstrable improvement with aspects of their physical, emotional and cognitive ability. Older people with mild to advanced forms of dementia appeared to benefit most. The sessions were enjoyable, and a real bond developed between the older people. | Not Reported. |
| Kallio *et al.,*  2021  Finland | To examine the effectiveness of a 12-week CT programme  for community-dwelling patients with dementia | Design  RCT.  Sample  Adult day care centres in Finland. Participants: (N=147) older individuals with mild to moderate dementia living at home and attending daycare.  Data Collection  Baseline, 3 months and 9 months.  Data Analysis  Statistical analysis.  Ethics Statement Provided. | Modification of cognitive remediation therapy (CRT) a Cognitive Training (CT) programme based on paper-and-pencil tasks with cognition as  a primary target, was designed for the FINCOG trial.  The intervention group (n = 76) received CT for 45 min twice a week while the control group (n = 71) attended day care as usual. | A 12-week CT programme provided in 14 adult day centres from September 2014 to March 2016. Trained psychology students administered CT under the guidance and supervision of a neuropsychologist. Two 45-minute sessions per  week during day care visits. Each session included cognitive exercises from four task categories: visuomotor, perceptual, conceptual, and interactive tasks (e.g., simple word or card games). Interactive tasks were included at the end of each session for up to 10 min to keep up the participants’ training motivation. | Base assessment CDR, MMSE, ADAS-Cog. A set of standard neuropsychological tests for executive and cognitive functioning. Task. Verbal concept formation and reasoning were assessed, and visuospatial reasoning was measured by means of Block Design, both from the Wechsler Adult Intelligence Scale, Fourth Edition (WAIS-IV). PWB scale. | No differences between the intervention and control group in the participants’ baseline characteristics: demographics (age, sex, and education),  self- or proxy-rated functional characteristics (living alone, daily activities, self-rated  psychological well-being), and clinical characteristics (dementia diagnoses, CDR, MMSE, comorbidities, and number of medications). | Training was tailored according to participants’ cognitive abilities, and accordingly implemented either in small groups of two to four people or individually when needed. The difficulty level was tailored during the sessions, but it was not automatic (as in computerized training). |
| Kwon *et al.,*    2020  Korea | To evaluate the effect of cognitive training programs on the progression of dementia in patients with early stage ADD at the day care center. | Design  Retrospective study.  Sample  Patients N=119 in dementia screening program of Namyangju City public health center (Jan 2015- Dec 2018).  Data Collection Demographic and clinical information and medication history.  Data Analysis  Fisher’s exact test and Mann-Whitney U test, and Student’s t-test.  Ethics Not stated. | Cognitive Training Programme. | All subjects were classified into two groups. 43 patients received both cognitive training and standard clinical care (case-subjects). 76 patients received only standard clinical care (control-subjects). Cognitive training was 3 hours a day, 5 days a week for 12 months at each day care center. A clinical neuropsychologist Instructor was present. Programs included attention training (i.e., paper or computer assisted attention training), memory training (i.e., training in the recall of a list and remembering the location of objects in the room), visuoconstruction training (i.e., drawing various things and changing blocks), physical training (i.e., massed calisthenics), occupational training (i.e., creative activity such as drawing or knitting), and speech training. | All patients underwent physical and neurological exam. Structured Attendance and compliance rate for cognitive training was not monitored.  MMSEDS, CDR, and CERAD) considered at baseline. Follow-up neuropsychological tests MMSE-DS, CDR, and sum-of-boxes (CDR-SOB)] were conducted 12 months after baseline and were compared between the two groups. | As compared to case-subjects (n=43), the MMSE-DS and CDR-SOB scores were significantly worse at 12 months in the control-subjects (n=76). A statistically significant difference between the two groups was observed due to changes in MMSE-DS (p=0.012) and CDR-SOB (p<0.001) scores. Multivariable logistic regression analysis showed that the cognitive training program (odds ratio and 95% confidence interval: 0.225, 0.070–0.725) was independently associated with less progression of ADD.  Application of a cognitive training program at a day care center in addition to usual standard clinical care showed significant benefit in terms of cognitive function in early ADD patients. | Not Reported. |
| Lancioni *et al.,*  2015  USA | To assess a program developed for helping patients with moderate Alzheimer’s disease engage in computer-mediated verbal reminiscence. | Design  Pilot Study.  Sample  Participants (N=16) with diagnosis of moderate Alzheimer’s disease.  (6 original program group  10 modified program group).    Data Collection  Each program version was introduced according to a nonconcurrent multiple baseline design across participants.  Data Analysis  Statistical analysis.  Ethics  Ethics Statement Provided. | Centers for persons with  AD and other dementias in self-care, leisure and occupational activities.  Also, various  periods of the day spent sitting with persons in  similar conditions in a fairly passive manner with marginal staff intervention. | Computer-aided sessions as well as baseline and control sessions were carried out. Sessions lasted 5 min. 6 participants used the original program version with the computer showing a virtual partner posing questions and providing attention and guidance. 10 used a slightly modified program version with the computer presenting photos and videos and encouragement to talk as well as attention and guidance. Typically, two or three computer-aided sessions occurred per day per participant (i.e., sessions were carried out on an individual basis). Control sessions were scattered through the intervention phase. Sessions were video-recorded and then scored by a research assistant. | Baseline phase included 2 or 4 sessions per participant. The intervention phase included 80–117 (M = 97) sessions for the participants using the first program version and 73–122 (M = 99) sessions for the participants using the second program version. Measures recorded: microswitch activations and verbal engagement; reminiscence. | The results showed that 15 participants (five of those using the first version and all of those using the second version) had a clear and lasting increase in verbal engagement/reminiscence during the intervention sessions with the program. Those 15 participants had mean percentages of intervals with verbal engagement/reminiscence below 10 during baseline and between about 45 and 75 during the intervention. | In the modified program version, the computer presented photos and videos and providing personalization to talk as well as attention and guidance. |
| Lin *et al.,*    2011  Taiwan | To examine the impact of an adaptive LRP on perceived QOL in elders with mild to moderate dementia at a day care center and assess the effect, if any, of different cognitive impairment levels  on intervention outcome. | Design  Pre-experimental, one-group pretest-posttest  Sample  Ever Green Adult Day Care Center, Taiwan. Participants (N=7) with mild to moderate cognitive impairment.    Data Collection  The Medical Outcomes Study 36-Item Short Form Health Survey (SF-36) was used to collect the data on changes in perceived QOL. Pretest data were collected 1 day before the 2-week intervention, and posttest data were collected 1 day after the 2-week intervention.  Data analysis SPSS.  Ethics Statement Provided. | Revised Life Review Programme  LRP-TW  Erikson’s life stages- Each session had a specific life theme in sequence, activities: singing lullaby songs, drawing lots, playing with traditional toys (e.g., bamboo dragonfly, catapult, sandbag, etc.), a traditional  puppet show and opera, listening to or watching old records and movies, doing garden activities, and enjoying the traditional tea ritual. | A revised version of LRP (LRP-TW) administered in 10 successive sessions over 2 weeks. Two to three staff from the center assisted. One session 60-minute session each weekday during the intervention period. Each session had a specific Erikson’s life stage theme in sequence, plus the first session as an introduction and the last session as a celebration. This framework guided the selection of relevant activities on the basis of how well each activity evoked memories related to target themes/ matched the life stage targeted. | 36-Item Short Form Health Survey (SF-36) was used to collect the data on changes in perceived QOL. | Two participants did not finish interventions because of family issues. Seven completed the intervention, and data was analyzed (mean age = 81 years, SD = 5.45 years, range = 70Y87 years; five women and two men; Table 3). All participants could read. Two had no schooling. The major cognitive impairment was memory loss. More than half of the participants (6 of 7) showed higher levels of QOL on the posttest than on the pretest, although improvements were not statistically significant.  LRP-TW may be an effective intervention to motivate elders with mild to moderate dementia to engage in early-age leisure activities and recall past events. | LRP-TW was adapted from the original LRP version and compressed into a short format that was administered in 10 successive sessions over 2 weeks. |
| Lorusso *et al.,*  2018  USA | To investigate the evidence regarding the use of multisensory environments (MSEs) as treatment for Behavioral and psychological symptoms of dementia (BPSD). | Systematic Literature Review.  Sample  Studies relating to participants predominantly 65 years and older with a diagnosis of moderate-to-severe dementia.  Data collection  PICO framework  Databases: PsycINFO, Web of Science, ERIC, PUBMED and Cinahl databases. Hand-searched documents.  Years published 1990-2015. | Multisensory Environments  -articles that incorporated at least three sensory-based items including ergonomic virbroacoustic furniture, bubble tubes, color-changing lights, music, and fiber optics. | RCT studies compared the effects of eight MSE sessions with control sessions. Some studies offered sessions at regular intervals during the week, same time daily and same duration each week. One study administered therapy intermittently. Some studies administered the MSE therapy specifically when the BPSD occurred. Approaches to data collection varied between 2 weeks to 1 year. MSE intervention sessions lasted between 8 and 40 minutes. None of the studies evaluated demonstrated whether the MSE therapy specifically addressed either under- or over-stimulation. | Behavior and/or mood were the most commonly assessed outcome variables across the 12 studies. Interact and Interact Short, Cohen-Mansfield Agitation Index (CMAI), Neuropsychiatric Inventory Nursing Home (NPI-NH), Cornell Scale for Depression in Dementia (CSDD), Psychotic Behavior Assessment Record (PBAR), Daily Observation Scale (DOS), and Clinical Global Impression-Improvement (CGI-I). | Generally, positive results regarding the impact of MSE therapy on BPSD were reported: A decrease in the number of BPSD incidences, positive changes in mood/behavior, and positive changes in engagement. Results on long-term effects were mixed, as some found that observed positive effects did not last significantly beyond the treatment sessions. One study demonstrated long-term benefits of MSE therapy at 12 weeks after treatment. Three studies examined how participants responded to the MSE equipment: one in a qualitative self-reporting format and two with an observational tool. | Not Reported. |
| Maeda *et al.,*  2016  Japan | To investigate a satisfaction index of dementia patient for emotional stimuli (Emotional Satisfaction Index [ESI]). | Design  Letter to the editor.  Sample  No mention of day services  Participants (N=9) with dementia diagnosis.  Data Collection  Measurement of satisfactory reactions.  Data Analysis  Not stated.  Ethics Not stated. | Dramatic Emotional Therapy | Emotional programs are carried out for approximately 1 h for dementia patients who visit the day care center at a psychiatric hospital for dementia patients.  Dramatic emotional therapy, Chorus with guitar,  volleyball using balloon, line drawing for coloring, karaoke, bowling game, watching TV program of popular song. | ESI is measured by summation of positive and negative satisfactory reactions of patients during an emotional program. | Music Chorus with guitar and volleyball using a balloon received positive reactions, but line drawing for coloring, karaoke and a bowling game received low scores,  Watching TV program of popular song received minus scores.  ESI could be applied for evaluating personal rehabilitation for dementia. | Not Reported. |
| Moorman *et al.,*  2017  USA | To evaluate the effects of diffused lavender on the frequency of Bis, defined as a composite of [RW], [AGT], [ANG], and [ANX] in an adult day care center. | Design  Quasi-experimental, pre-post.  Sample  Private nonprofit adult day care center for patients with dementia. Participants (N=23) elderly patients older than 65 years of age which a clinical diagnosis of dementia, who require daytime monitoring.  Data Collection  Observations/Recording.  Data Analysis  SAS version 9.3.  Ethics Statement Provided. | Lavender aromatherapy. | This was a pre-post quasi-experimental study conducted over a four-month period between June and September of 2013. Lavender aromatherapy was implemented immediately after a two-month pre-intervention observation phase. Lavender essential oils (Young Living Essential OilsTM, Lehi, UT) were diffused using the Advanced Essential Oil Diffuser (Abundant Health, LLC., Spanish Fork, UT) in the day care center common area for 20 min twice a day, once in the morning and once in the mid afternoon during active clinic days. Lavender aromatherapy twice a day for 20 min during a two-month period during active  clinic days. | During the pre- and post-intervention phases, Bis recorded using the Behavior Intervention Monthly Flow Record (MED-PASS Inc. and Heaton Resources, Dayton, OH). Behavioral issues recorded pre- and post-intervention. The observer was a certified nurse assistant with training and experience in using the form and documenting information. Bis that occurred during the study period were addressed as per center protocol with either one on one staff time or use of diversional activities appropriate for each behavior. Adverse events to the use of diffusers were noted. | Bis included the combination of restlessness/wandering, agitations, anger, or anxiety. There was no significant difference on frequency of Bis between pre-intervention and postintervention periods (p.06). Use of diffused lavender did not show statistical difference in reducing the frequency of other behaviors (restlessness/  wander, anger, anxiety), the study population may have been too small to find a difference; a trend towards decreased Bis was seen. | Not Reported. |
| Noone & Jenkin,  2018  UK | To explore how people with dementia experience participating in community-based gardening  initiatives. | Design  Pragmatist methodological perspective, drawing upon phenomenology and action research.  Sample  Daycare centre for people with dementia. Participants (N=6) centre attendees.  (N=3) day centre staff.  Data Collection  Mixed methods. Qualitative interviews and researcher observations.  Data Analysis  Thematic analysis.  Ethics Statement Provided. | Gardening session. | Weekly gardening sessions for people with dementia (n=6). Weekly session over six weeks. A cyclical process was fundamental to the design of the gardening programme, as each session formed an action research cycle. The feedback obtained from participants at the end of each session informed the planning of the subsequent session, driving the project further towards the ‘optimal solution’ to the research problem. | The small sample size and qualitative, inductive data collection tools associated with phenomenological studies enabled the researcher to form relationships with the participants and immerse themselves in the data, contributing to a deeper interpretation of the group’s experiences of the activity. | Three themes identified: the role of gardening in encouraging expressions of identity; demonstrations of agency; and the development of community. Gardening offered participants the opportunity to express elements of their identity and character. Improved level of engagement. Shared knowledge of gardening; sense of control and involvement. Enjoyment from the opportunity to work autonomously and make free choices. A sense of freedom from the restrictions impinging upon their everyday lives creating a sense of agency. Opportunities for social interaction and create a new social dynamic; confidence to break out of social routine form new social groups and develop friendships. | Over the course of the gardening project, the researcher developed a relationship with each participant, facilitating the development of an understanding of the participants’ verbal and non-verbal cues, and creating a mutual trust with the participants that enabled them to feel comfortable communicating their level of willingness to participate in a particular activity. |
| Novy,  2018  Canada | To develop a life story project for older adults living with dementia, to increase opportunities to connect with one another in meaningful ways. | Design  A report on the project combining clinical vignettes from one life story performance, along with observation, reflective practice and participant questionnaires.  Sample  From a nonprofit adult day centre that provides activity programs for adults living with dementia, participants (N=12) storytellers participated in the life story project.  Data Collection  observation, reflective practice and participant questionnaires  Data Analysis  Not stated.  Ethics not stated. | Life story work. | The life story project over its two- year lifespan was a complex intervention, that included a phase of individual work for the purpose of recording a life story, followed by an interactive performance of this story in a group setting. Facilitated by 12 storytellers over two years. Life story recorded and performed by utilizing a narrative, centering the person, picturing the story, recruiting an audience, dramatizing the story and performing the life story. | Video analysis of conversations, observations, case materials from the life story and vignettes with discussion. | PWD helped to reveal participant personhood and present opportunity for individuals to connect with members of their community in new ways and alleviate some of the social and emotional isolation. PWD helped to build bridges to communication and connection among and between peers at the day program by strengthening peer relationships and restore a sense of identity in both the storyteller and their listener.  Performing the life story drawing from drama therapy and narrative  therapy. | Not Reported. |
| Osman *et al.,*  2016  UK | To explore the impact of Singing for the Brain. | Design  Qualitative.  Sample  Participants (N=20) -10 patient-carer pairs  Data Collection  10 semi-structured paired participant interviews (patient-carer).  Data analyses  Thematic.  Ethics Statement Provided. | Singing for the Brain. | Group activity combining reminiscence therapy and music, involving a musician, PWD and their caregivers. During a session, carer–patient pairs gathered in a large circle and followed instruction from the musician. The session started with warm-up exercises for voice and body before moving on to singing familiar songs that followed a different theme each week.  Attendees were provided with song sheets. Songs were sung in unison accompanied by the musician or in rounds with harmonies. Depending on access to equipment, attendees could use percussive instruments. At the beginning and end of each session, there was an opportunity for refreshments and time to ersonali. | Not stated. | Six themes identified; (1) Social inclusion and support  (2) A shared experience  (3) Positive impact on relationships  (4) Positive impact on memory  (5) Lifting the spirits  (6) Acceptance of the diagnosis.  Social inclusiveness and improvements in relationships, memory and mood were found to be especially important to participants. As well as enjoying the sessions, participants found that attending Singing for the Brain helped in accepting and coping with dementia. | Not reported. |
| Olsen *et al.,*  2019  Norway | To systematically register behaviours related to engagement in a group animal assisted activity intervention. | Design  RCT.  Sample  Out of 90 eligible NHs, 10 adapted NHs for PWDs and 16 (out of 108) adapted DCCs for home-dwelling PWDs. Participants (N=21) NH and (N=28) DCC having dementia or a cognitive deficit.  Data Collection  Video recording done in week 2 and 10.  Data analysis  behaviour coding software Solomon Coder, SPSS  Ethics Statement Provided. | Animal assisted activity (AAA). | 30-min AAAs sessions in groups of 3-7 participants twice per week for 12 weeks, led by a qualified dog handler. A protocol was followed. The intervention had a strict design standardized as far as possible. For each session, the participants were randomly seated in a semi-circle. Every session  started with a greeting round, where each participant got to pet the dog and give it treats. The handler then started the different activities. A health care worker was present during all sessions. The dogs were kept on/off lead, depending on interaction. No dogs were forced to do anything they were not comfortable doing. | MMSE Clinical Dementia  Rating (CDR) Scale, Sociodemographic characteristics CDR Scale Video-recordings using a camera Sony HXR-NX30E.  An ethogram catalogue of behaviour descriptions. | Four of the NH participants did not have a dementia diagnosis, and the mean MMSE for these participants was 15.3 (SD=6.7, range: 7–23). For DCC participants, the mean MMSE for the eight participants without a dementia diagnosis but with a MMSE score was 18.4 (SD=6.2, range: 8–26) None of the DCC participants were assessed as having severe dementia, majority had moderate dementia (53.6%).  Mean values showed that actions towards the dog, such as observing it, smiling, talking to it or petting it, were the behaviours with the longest duration in AAA sessions in both populations. | Even though the sessions were designed to follow a protocol, they could also be individually tailored. No activities were mandatory, and the sessions included activities that naturally occurred between the participants, and between each participant and the dog. |
| Olsen *et al.,*    2016  Norway | To examine whether animal assisted activities would influence factors related to the risk of fall accidents, with balance and QoL. | Design  A prospective and cluster-randomized multicentre trial with a follow-up study.  Sample:  16 day-care centers. Participants (N=80) 65 years or older and had either a diagnosis of dementia or a cognitive deficit. Intervention group (N=42) control group (N=38).  Data Collection:  Pre-Test, when the intervention finished, and at a three-month follow-up.  Data Analysis  SPSS for descriptive and inferential (ANOVA).  Ethical statement provided. | Animal Assisted Activity (AAA). | AAA led by qualified dog handler, 30-minute sessions twice a week for 12 weeks which followed a protocol to ensure consistency between the intervention sessions. The intervention had a relatively strict design and was standardized as far as possible, despite the fact that one of the study objectives was to see whether it was possible to measure effects when AAAs occurred in a realistic setting with a representative sample of participants and different dog teams. | Berg Balance Score, Quality of Life in Dementia Scale, Clinical Dementia Rating scale.  (Table 2). No effect was found at follow-up, even though the improvement experienced by the intervention group remained constant after T1. | Berg Balance Score for participants in the animal assisted activity scored significantly lower than those in the control group. Findings revealed a significant difference between the groups in the pre-test regarding BBS score, as participants in the AAA group scored significantly lower than those in the control group (p ¼ 0.01). The mean score on the BBS for the AAA group was 41.55 at T0, with an increase to 44.71 at T1, and a score of 44.28 at T2. The control group scored 45.31 at T0, 45.50 at T1, and 46.57 at T2. On balance, the AAA intervention was a significant positive effect from T0 to T1 (p ¼ 0.03).  The animal assisted intervention had a significant positive effect. | All sessions followed the main protocol, but they were individually tailored to each participant based on the care workers’ knowledge of the participant. Hence, none of the AAAs was mandatory during the sessions, and the sessions included activities that naturally occurred between the participants, and between each participant and the dog. |
| Padilla *et al.,*  2013  Spain | To explore the effectiveness of an intervention program for reducing the frequency of wandering behavior of a patient with dementia, using different non-invasive and non-restrictive strategies (environmental and cognitive-behavioral). | Design:  Case Report.  Sample:  80-year-old man with dementia diagnosis.  Data Collection:  MMSE, RGDS, CAMCOGR, BBDLAS, CDLAS, CSDD, AS.  Data Analysis:  A single case analysis.  Ethics  Statement of ethics approval not provided. Legal guardian provided written informed consent. | Environmental (subjective barriers), cognitive /behavioral (cognitive training with differential reinforcement) and combined (subjective  barriers + cognitive  /behavioral). | 33 sessions of four hours.  Environmental intervention the exit door was altered (eight strips of black tape were placed 25cm from the exit door with 4 cm distance between and four other strips placed on the glass door 25 cm from the floor. Cognitive/behavioral intervention consisted in differential reinforcement of behaviors while working with the patient on different types of language, memory, and attention tasks which were used to distract the persons attention from the door. | Algase Scale (AS) used to evaluate wandering behavior.  The frequency of escape attempts, measured throughout the study, was the dependent variable,  operationally defined as “the number of times the Center’s glass exit door was approached within the distance of the alarm sensor (approximately 2 m).” | A decrease In wandering behavior frequency in the person from an average of 37 escape attempts to an average of one or two a day.  The person participated in most activities and interacted more with the people around him in the center. | Not Reported. |
| Peeters *et al.,*  2016  Netherlands | To develop the conceptual design and design rationale of the Music ePartner. | Design:  A human-centred design method, called situated Cognitive Engineering,  developed specifically to support the design of intelligent human-computer interaction.  Sample:  Participants (N=5) PwD accompanied by a close relative  Data Collection:  Observations  Questionnaires  Scoring forms  Semi-structured interviews    Data Analysis  Not stated  Ethics: Statement of ethics approval not provided. Informed consent obtained from participants. | Music application prototype. | Person with dementia and relative paired to use the prototype during an evaluation day. | Formative evaluation investigated the process of how users interacted with the design through observations and interviews. This evaluation was diagnostic in nature, focusing on the prototype’s usability, acceptability, and desirability, so as to refine the initial requirements baseline.  Evaluated against three functionalities–- music collections management, personal multimedia slide show and personal music picture album. | Positive outcomes:  Reacted strongly to the music and mood improved.  Unable to use table himself.  Started talking about old memories and was in a more active mood.  Knew many of the lyrics and evoked memories.  Improved engagement and interaction.  Recognised people in photos.  Enjoyed the music.  Chatted about the music and other topics. | To explain the navigation through the different screens of music collections management functionality, the researcher used an analogy of a house with different rooms, in which buttons represent doors that enable navigation between the rooms. |
| Riley-Doucet and Dunn    2013  USA | To examine the feasibility of using a Multisensory environment (MSE) as a nonpharmacological intervention to reduce agitation among people exhibiting behavioral and  psychological symptoms of dementia (BPSD) within an adult day care centre. | Design: Quantitative Pilot, within-subjects, repeated measures design.  Sample: Participants (N= 8) service users and (N= 4) caregivers  Data Collection: medical record review for demographics along with questionnaires x 2. Observational data.  Data analysis:  PASW version 18.0  Ethics Statement provided. | Multisensory environment. | 4-week period (average 5 times a week and average 25 minutes per session) utilizing the following sensory equipment, aroma diffuse, chase light string, evening breeze, fibreoptic string light, plasma ball, solar effects projector, stereo and speakers (music) and vibrating tube. | An observation record was used to record the length of time the older adults spent in the MSE and their overall enjoyment of the multisensory environment session.  The Agitated Behavior Scale was used to collect observational data of BPSD.  Caregiver Exit Survey to assess caregivers’ satisfaction with using the intervention. | Enjoyed multisensory intervention.  Connected with caregivers in multisensory environment.  Responded positively to multisensory intervention. | Not reported. |
| Rokstad et al.,  2019  Norway | To explore and compare the experiences and outcomes of day care services designed for people with dementia as described by day care attendees and their caregivers in Norway and Scotland. | Design: Qualitative descriptive  Sample:  Norway–- recruited from the ECOD study (Rokstad et al., 2014). (N= 17) people with dementia and caregivers recruited from (n=6) centres.  Scotland–- (n= 19) people with dementia and (n=15) caregivers from (n=6) centres participated.  Data Collection: interviews.  Data Analysis: Thematic Analysis. | Reporting on the experiences of people with dementia attending day care services and their main caregivers and the outcomes they reported. | Not described | Not stated. | Four themes reported:  The experience of day care (from reluctance to acceptance and enjoyment, day care structures the days, conversations, mealtimes and meaningful activities)  The importance of relationships  Outcomes for people with dementia  Outcomes and experiences of caregivers  Commonalities and some important differences were found across the data from the two countries, and these are presented within these four themes. | Not reported |
| Rylatt  2012  United Kingdom | To evaluate the use of creative therapy with people with dementia. | Design: Quantitative  Sample: Participants (N= 37) participants with dementia.  Data Collection: Data collection tool designed by the project lead and the clinical governance support team.  Data analysis:  predictive analytic software was used to input and analyse the data, and descriptive results were produced by an information and project support officer from the clinical governance team.  Ethics Statement provided. | Creative therapy activities including dance, drama, music and movement. | Implemented over an eight-week period, three times a week, with each session lasting approximately 30 minutes. | Data collection tool was designed to evaluate information on attendance, activities (including warm-up and session closing strategies) and outcomes of the creative therapy sessions in relation to creative self-expression, communication, pleasure and enjoyment, and general engagement.  Staff running the activity recorded the degree of improvement seen in the participant during each session. | Improved social skills and connection with those around them.  Improved communication.  Pleasure and enjoyment.  Greater engagement. | Not reported. |
| Salotti *et al.*  2013  Italy | To evaluate the efficacy of a cognitive rehabilitation intervention performed in an Alzheimer’s Day Care Centre for 12 months on patients with Alzheimer’s type dementia with moderate cognitive impairment. | Design: Quantitative  Sample: n=9  (Experimental group = 4, Control group= 5).  Data Collection: Milan Overall Dementia Assessment and the Mini-Mental State Examination were used for the cognitive domain.  Geriatric Depression Scale was used for the affective sphere.  Data analysis: Statistical analysis using SPSS v15.  Ethics not Stated | Reality orientation therapy (ROT) and cognitive training (CT) constituted cognitive rehabilitation for the experimental group and the control group has aspecific stimulation. | All participants underwent a series of aspecific group stimulations 3 days a week.  The experimental group also underwent cognitive stimulation based on a combination of two therapies — ROT and CT — three times a week. | Assessments done prior and post each session 1 hr session (3 x week) provided by a psychologist expert in neuropsychology.  Data from the ongoing assessments of Milan Overall Dementia Assessment and Mini Mental State Examination tests (for cognitive function) and Geriatric depression score (affective) were statistically analysed | After 9 months, cognitive function was maintained by experimental group | Bespoke programme developed for memory, attention and language functions using various methods and aids. |
| Shoesmith *et al*.  2020  UK | To assess the feasibility and acceptability in delivering a newly developed therapeutic, person-centred visual art intervention for people with dementia attending a day care centre or residing in an assisted living facility. | Design: mixed-methods, quasi-experimental, pre/post design.  Sample: N = 15 people participated in two groups.  5 older people with 2 arts facilitators in day care centre  6 older people plus 2 facilitators in assisted living  Data Collection: Quantitative and qualitative.  Data analysis: Thematic analysis  Ethics Statement provided. | Intervention: structured, person-centred session. Each week = different activity inspired by a different theme, involves a variety of materials (e.g. paints, ink, pencils, fabric, 3D materials) and techniques (e.g. brush, paint roller, pipettes) aimed towards stimulating participant choice and independence. | The intervention six weekly, one- hour sessions, co-facilitated by two trained facilitators.  Intervention = structured, person-centred session plans, as the activities are strengths-based (focusing on internal strengths) and process-orientated (focusing on the creative process), or performance oriented (focusing on creating an aesthetically pleasing product).  Weekly different theme, different activity variety of materials e.g., paints, and techniques session involved introductions, warm-up exercises, the artistic activity and final debriefing before finishing group. | Quantitative assessment of the impact of the intervention on QoL, ADL and social functioning at three time points (T0: baseline, T2: post-intervention, T3: one-month follow-up). Outcome measures were rated on established test instruments.  Qualitative interviews (interviews at T1 and T2). | Quantitative data suggests enhanced social function and quality of score post interventions.  5 themes from qualitative data reflected the acceptability and feasibility of the intervention.  3 themes identified the successful outcomes of the intervention such as participant choice,ersonalizatin & mentally stimulating activities. | N/A newly developed intervention. |
| Smith *et al*.  2020  UK | To explore how tablet computers might encourage participation in enjoyable activities by people with moderate levels of dementia and to consider how such technologies might be incorporated into the repertoire of activities provided through day care settings. | Design: Qualitative, focused visual ethnographic approach.  Sample: n= 12 participants attending a community day care centre  and  n=9 supporters (volunteers (4), researcher (1) manager (1), staff (2), mental health student on placement (1).  Data Collection: video recording.  Data analysis: video analysis.  Ethics Statement provided. | Technology facilitated group activity sessions took place twice a week for a period of four weeks and all were video recorded. The researcher uploaded the tablet computers with familiar activities already enjoyed by members that appeared to effectively transfer from their traditional form to technology formats, including dominos, solitaire and jigsaws. | New interactive applications were also uploaded including the keyboard, guitar and drum kit. Quantity of applications increased as researcher became more familiar with the group and individual preferences became apparent. The flexibility of the devices and the plethora of available applications enabled the personalizationn of the devices to the voiced preferences of the group.  The flexibility of many of the applications enabled levels of difficulty to be adjusted to the person’s capabilities. | Video analysis, multimodal interactional analysis | Majority of people with dementia found the technology an effective means of participating in enjoyable activities.  Maintaining focus on retained strengths and abilities enabled the group overall to meet and often exceed their own and others perceived capacity to participate.  Analysis confirmed the importance of enjoyment of activities ‘in the moment’ and the need for those supporting people in the moderate stages of dementia to acknowledge and work with this. | Not reported |
| Sánchez-Valdéon *et al*.  2019  Spain | To determine whether canine-assisted therapy maintained or changed the quality of life of people with severe Alzheimer-Type Dementia (ATD) over time (1 year). | Design: Quasi-experimental study, with a simple pre-post case series design.  Sample: n= 10 service users presenting with severe or very severe cognitive decline.  Data Collection: Quality of Life in Late-Stage Dementia (QUALID) scale, data collected at 3 intervals.  Data analysis: SPSS v24  Ethics Statement Provided | Canine therapy.  The dog used for the therapy was a Labrador specifically trained by a canine specialist. It had been socialized, had a stable, friendly nature, was clinically healthy, and had been properly vaccinated against infectious diseases. The intervention was carried out by a professional trained in canine-assisted therapy. | Canine therapy with dog and skilled professional 30 minutes per week for 12 months.  A skills programme included a mix of guided motion exercises, activities and animal to human interaction for improving affective state. | The measurement instrument employed was the Quality of Life in Late-Stage Dementia (QUALID) scale. | At end of the therapy, 100% of participants showed an improvement in physical, behavioural, and psychological aspects.  The total scores of the QUALID scale for the three different evaluation times (before the therapy, at 6 months and 12 months after starting) after the canine-assisted therapy intervention were smaller and less dispersed.  The total score for the QUALID scale decreased significantly (p< 0.05) at 6 and 12 months after starting therapy. An analysis by item revealed that the scores of all fell during the course of the therapy.  Study provides evidence of the significant benefits of canine-assisted therapy for quality of life in people with Alzheimer’s disease. | Not reported |
| Soler *et al*.  2015  Spain | Pilot studies applying a humanoid robot (NAO), a pet robot (seal) (PARO) and a real animal (DOG) in therapy sessions of patients with dementia in a nursing home and a day care centre  aim to test the effect of introducing these in the therapeutic sessions for patients with dementia in relation to behaviour changes, apathy and quality of life. | Design: 1) Nursing home = controlled clinical trial of parallel groups, randomized by blocks (living units) and stratified by dementia severity. 2) Day Care centre = pre-test/post-test design.  Sample:  Nursing Home: Phase 1 n=101 Phase 2 n= 110  Day Care Centre: Phase 1 n=20 Phase 2 n=17  Data Collection: validated scales.  Data analysis: statistical analysis using Stata software.  Ethics Statement provided. | Nursing home–- Phase 1) a humanoid robot (NAO), who is able to use oral language (phrases previously recorded) and move like a human; an animal-shaped robot (PARO), who does not use oral language but sounds and moves like an animal; and with conventional therapy (CONTROL) (Phase 2) a trained dog (DOG); an animal-shaped robot (PARO); and with conventional therapy (CONTROL)  Day care center, all patients received therapy with NAO (Phase 1) and PARO (Phase 2). | Therapy sessions were held 2 days per week for 3 months. All therapeutic sessions were conducted by the same therapist, with the same structure as the other therapeutic programs, at the same time of day and for the same duration of time (30–40 min).  The therapists were certified occupational and physical therapists, and neuropsychologists, they received instructions on the implementation and possible uses of robots and animals as they had no previous expertise in this area.  The animal therapists and robot engineers did not participate in the therapy; they monitored session from one side of the room, out of the patients’ view. Session guides were written and followed in every session. | Evaluation, at baseline and follow-up, carried out by blind raters using: the Global Deterioration Scale (GDS), the Severe Mini Mental State Examination (sMMSE), the Mini Mental State Examination (MMSE), the Neuropsychiatric Inventory (NPI), the Apathy Scale for Institutionalized Patients with Dementia Nursing Home version (APADEM-NH), the Apathy Inventory (AI) and the Quality-of-Life Scale (QUALID).  Statistical analysis included descriptive statistics and non-parametric tests performed by a blinded investigator. | The main findings were: (Phase 1) improvement in the NPI irritability and the NPI total score; (Phase 2) No differences were observed at follow-up. Participants showed improvements in irritability and global neuropsychiatric symptoms after participating in sessions with the humanoid robot, but not after sessions with the animal-shaped robot. | Not reported |
| Thompson & Fletcher  2019    Canada | To examine the perceived effects that an adult day program had on individuals with dementia and their caregivers, from the perspective of care providers at an adult day Program. | Design: Qualitative design.  Sample: n=7 adult day program care providers.  Data Collection: interviews.  Data analysis: thematic analysis.  Ethics Statement provided. | Difficult to say as it was a day care programme in general which included physical care provision but did also include the use of social interactions and the development of social relationships which in themselves could be classed as PSI. | Not specifically outlined all within the whole programme, so not explicate as to when/how specific psycho-social interventions were focused upon. | Thematic analysis was used to analyze the data. | The adult day program care providers identified several benefits of adult day programs for individuals with dementia and their caregivers. Two themes emerged, including (1) the role of care providers and (2) time to breathe Specifically re PSI, maintaining dignity and self-worth, social interactions and forming social relationships, and engagement in physical activities from theme 1 (theme 2 did not refer to PSI effects/interactions) | Not reported |
| Van Bogaert et al.  2013  Belgium | To examine the effects of individual thematically based reminiscence sessions based on SolCos model for older adults with dementia (probably Alzheimer’s) | Design: pre-test post-test intervention  Sample: n=82 (42 in control group, 42 in intervention group)  Data Collection: validated assessment scales to evaluate cognition and behaviour  Data analysis: statistical analysis  Ethics statement provided. | Structured reminiscence sessions based on SolCos reminiscence model. | 6-8 in total (average 7.4) over 4 weeks, 1 facilitator | Pre and post interventions period tests for validated assessment scales for cognition and behaviour evaluation.  Mini- Mental State Examination (MMSE)  Frontal Assessment Battery (FAB)  Neuropsychiatric Inventory (NPI)  Geriatric Depression Scale (GDS-30  Cornell Scale for Depression in Dementia (CSDD) | Positive effects associated with individual thematically based reminiscence on well-being such as depressive symptoms and cognition of participants.  Better MMSE and GDS in intervention group than control, mild and mod AD had better GDS and better MMSE for mod AD | Not reported |
| Thompson & Fletcher  2019    Canada | To examine the perceived effects that an adult day program had on individuals with dementia and their caregivers, from the perspective of care providers at an adult day Program. | Design: Qualitative design.  Sample: n=7 adult day program care providers.  Data Collection: interviews.  Data analysis: thematic analysis.  Ethics Statement provided. | Difficult to say as it was a day care programme in general which included physical care provision but did also include the use of social interactions and the development of social relationships which in themselves could be classed as PSI. | Not specifically outlined all within the whole programme, so not explicate as to when/how specific psycho-social interventions were focused upon. | Thematic analysis was used to analyze the data. | The adult day program care providers identified several benefits of adult day programs for individuals with dementia and their caregivers. Two themes emerged, including (1) the role of care providers and (2) time to breathe Specifically re PSI, maintaining dignity and self-worth, social interactions and forming social relationships, and engagement in physical activities from theme 1 (theme 2 did not refer to PSI effects/interactions) | Not reported |
| van Haeften-van Dijk, *et al*.  2017  Netherlands | To investigate whether community-based (CO) day care with carer support according to the proven effective “Meeting Centres Support Programme” model is associated with higher satisfaction of people with dementia (PwD) and their informal caregivers (CG) and with a higher job satisfaction among care staff compared to traditional nursing home-based (NH) day care. | Design:  Part of implementation study new CO Day care centres specifically targeted Turkish and Moroccan immigrants.  Sample: n=35  Data collection: Questionnaire  Data analysis:  statistical analysis  Ethics Statement  Provided | User satisfaction of participants with dementia and their carers was measured with two questionnaires developed by Dr€oes *et al*. (2004) and Dr€oes *et al*. (2011) for the evaluation | Not described | Not stated | PwD were more positive about the communication and listening skills of staff and the atmosphere and activities at the CO Day care centre. Also, CG valued the communication with, and expertise of, staff in CO Day care higher, and were more satisfied with the received emotional, social and practical support. After the transition, satisfaction of staff with the work pace increased, but satisfaction with learning opportunities decreased. | Not reported |
| Westcott  2011  USA | A descriptive piece about the author’s time observing /volunteering in a respite care adult day care agency (not research) | Design: Observation and description of a group in morning sessions.  Sample: 1-6 clients, 1-3 staff.  Data Collection: Observational.  Data analysis: Narrative analysis. | Recreational groupwork included a variety of activities such as bingo, scrabble, word search and poems, initially aimed at one specific individual | Group not static but generally 2-6 male and female members & 5 to 95) with 1 to 3 staff. | Not really except a loose observation. | Anecdotally noted improvement in engagement. | Not reported |
| Alzheimer’s Disease (AD), Activities of Daily Living (ADLs), Adult Day Health Centers (ADHC), Algase Scale (AS) Barthel Basic Daily Life Activities Scale (BBDLAS), Cognitive Therapy (CT), Cambridge Cognitive Examination Revised (CAMCOGR), Cornell Scale for Depression in Dementia (CSDD), Crucial Daily Life Activities Scale (CDLAS), Emotional Satisfaction Index (ESI),Farm Based Day Care (FBDC), Instrumental Activities of Daily Living (IADLs), Mini-Mental State Examination (MMSE), Multisensory Environments (MSEs), National Institute of Neurological and Communicative Disorders and Stroke-Alzheimer disease and Related Disorders Association (NINCDS-ADRDS), Reisberg Global Deterioration Scale (RGDS), | | | | | | | |
